# Implications of Scientific Collaboration Networks on Studies of Aquatic Vertebrates in the Brazilian Amazon

**DOI:** 10.1371/journal.pone.0158413

**Published:** 2016-06-28

**Authors:** María Celeste Salinero, Fernanda Michalski

**Affiliations:** 1 Laboratório de Ecologia e Conservação de Vertebrados, Universidade Federal do Amapá, Macapá, Amapá, Brazil; 2 Programa de Pós-Graduação em Biodiversidade Tropical, Universidade Federal do Amapá, Macapá, Amapá, Brazil; 3 Instituto Pró-Carnívoros, Atibaia, São Paulo, Brazil; VU University Amsterdam, NETHERLANDS

## Abstract

The quantity of wildlife extracted from the Amazon has increased in the past decades as a consequence of an increase in human population density and income growth. To evaluate the spatial distribution of studies on subsistence and/or commercial hunting conducted in the Brazilian Amazon, we selected eight mid-sized and large-bodied aquatic vertebrate species with a history of human exploitation in the region. We used a combination of searches in the gray and scientific literature from the past 24 years to provide an updated distributional map of studies on the target species. We calculated the distances between the study sites and the locations of the research institutes/universities that the first and last authors of the same study were affiliated to. For the period of 1990 to 2014, we found 105 studies on the subsistence and/or commercial hunting of aquatic vertebrates in the Brazilian Amazon in 271 locations that involved 43 institutions (37 Brazilian and 6 international). The spatial distribution of the studies across the Brazilian Amazon varied, but over 80% took place in the northeast and central Amazon, encompassing three States of the Legal Brazilian Amazon (Amazonas, 51.42%; Pará, 19.05%; and Amapá, 16.19%). Over half of the research study sites (52.91%) were within 500 km of the research institute/university of the first or last authors. Some research institutes/universities did not have any inter-institutional collaborations, while others collaborated with eight or more institutes. Some research institutes/universities conducted many studies, had an extensive collaboration network, and contributed greatly to the network of studies on Amazonian aquatic vertebrates. Our research contributes to the knowledge of studies on the subsistence and/or commercial hunting of the most exploited aquatic vertebrates of the Brazilian Amazon, illustrates the impact that collaboration networks have on research, and highlights potential areas for improvement and the generation of new collaborations.

## Introduction

After habitat loss, direct human exploitation via hunting (for subsistence or commercial markets) is one of the biggest threats to sustainability in tropical regions [1–3]. Protein acquisition by local communities in tropical regions relies heavily on meat from wild vertebrates [4, 5], and some aquatic species, such as caimans [6, 7], freshwater turtles [8–10], manatees [11], and river dolphins [12], have a long history of human exploitation in the Amazon region. The use of these aquatic species is not only related to meat consumption but also to traditional medicine [13] and religious beliefs [7, 14], which in turn make the conservation of these species even more challenging.

Brazil has a territorial surface of 851 million hectares [15] and contains the largest protected area system in the world, which encompasses nearly 220 million ha [16]. The Legal Brazilian Amazon has an area of 510 million ha [17] that contains approximately 40% of the world’s remaining tropical forests [18], and hosts a large fraction of the Earth’s biodiversity.

As of December 2010, the Legal Brazilian Amazon had 307 Conservation Units (CUs) covering over 117 million ha. Of these, the majority were sustainable-use reserves (n = 196) while 111 were strictly protected [19]. Therefore, the majority of these reserves face the difficult challenge of balancing guaranteed access by local communities to natural resources whilst ensuring the persistence of biodiversity and ecological processes [20]. This is a delicate balance that requires the support of legislation [21], and the effectiveness of CUs in protecting biodiversity can be compromised by weak or inappropriate management responses [22] to external threats that undermine the main goals of the reserves [23, 24].

In addition, due to a combination of factors such as overexploitation, water contamination, habitat modification, and the invasion of exotic species, aquatic environments are one of the most globally endangered habitats [25], with a higher biodiversity decline than terrestrial ecosystems [26]. Aquatic environments in the Legal Brazilian Amazon are also home to important sources of protein that sustain most of the region’s local communities.

The quantity of wildlife extracted from the Amazon has increased over the past decades as a consequence of a rise in human population density, growth in income, and a greater participation of hunters in the market economy [27]. As a result, studies on subsistence and commercial hunting in the Amazon region have also increased in the past decades [3, 4, 6–9, 12, 27–29].

However, to our knowledge, no evaluation has been conducted of the spatial distribution of studies conducted on the subsistence and commercial hunting of aquatic vertebrates in the Amazon. Therefore, there is a need to understand the spatial distribution of studies that have been conducted to evaluate the impacts of hunting on aquatic species in such an important and diversity-rich region as the Brazilian Amazon. In addition, co-authorship networks are considered an important class of academic social networking [30], and have been the subject of intense interest in past decades as they can influence the behavior, motivation, and performance of scientific collaborations [31, 32].

We had two research objectives in this study. Firstly, to produce a map that showed where studies of the most overexploited Amazonian aquatic vertebrates had been conducted. Secondly, to explore the distribution of these studies in relation to the location of the research institutes and universities of the affiliated authors, and to examine how researchers cooperate with each other in a scientific network of collaborations. The outcomes of these two research objectives can contribute significantly to knowledge of the current state of studies on heavily exploited aquatic species in the Brazilian Amazon. Research institutes and universities will be able to know that they are contributing to a network of collaborations as well as strengthening their scientific relationships with other research institutes that have studied aquatic vertebrates in past decades. Researchers will also be able to identify potential areas where more effort should be exerted in studying Brazilian Amazon aquatic vertebrates. Therefore, this study will identify locations that have not been studied in terms of the subsistence and/or commercial hunting of aquatic vertebrates, and improve scientific collaborations between research institutes.

## Materials and Methods

### Species selection

We selected aquatic vertebrates that met the following criteria: 1) were distributed across Amazonia, 2) were used (historically or currently) for subsistence and/or commercial use, 3) were mid-sized or large-bodied and easily identifiable, and 4) were classified as Least Concern But Conservation Dependent, Data Deficient, or Vulnerable according to the International Union for Conservation of Nature Red List. We selected eight species that fully met these criteria. We chose two species of freshwater turtle (*Podocnemis expansa*, the South American river turtle and *Podocnemis unifilis*, the yellow-spotted river turtle), two species of caiman (*Melanosuchus niger*, the black caiman and *Caiman crocodilus*, the spectacled or common caiman), two species of manatee (*Trichechus inunguis*, the South American manatee and *Trichechus manatus*, the West Indian manatee), and two species of river dolphin (*Sotalia fluviatilis*, the Tucuxi and *Inia geoffrensis*, the Boto or pink river dolphin).

### Compilation of studies

We reviewed the available literature by focusing on the subsistence hunting and commercial use of aquatic vertebrates in the Legal Brazilian Amazon. We first conducted searches of the ISI Web of Knowledge, SciVerse, Scopus, Google Scholar, and Scielo using combinations of the terms “Amazon”, “wildlife”, “conflict”, “hunting”, “subsistence hunting”, “consumption of animals”, “conservation unit”, “indigenous reserves”, “Chelonia”, “Crocodilia”, “Sirenia”, “Cetacea”, “Pará”, “Amapá”, “Amazonas”, “Acre”, “Roraima”, “Rondônia”, “Mato Grosso”, “Maranhão”, and “Tocantins”. Searches using equivalent terms in Portuguese and Spanish were also conducted to cover the period from 1990 to 2014. We conducted additional searches of the literature cited in each article obtained in our survey and from reports of the Instituto Chico Mendes de Conservação da Natureza (ICMBio) and the Instituto Brasileiro do Meio Ambiente e dos Recursos Naturais Renováveis (IBAMA) that are available on the internet. The gray literature (i.e., conference abstracts, reports, graduate theses, MSc theses, and PhD dissertations) was also searched using the same combination of terms in Google and Google Scholar.

**Fig 1:**
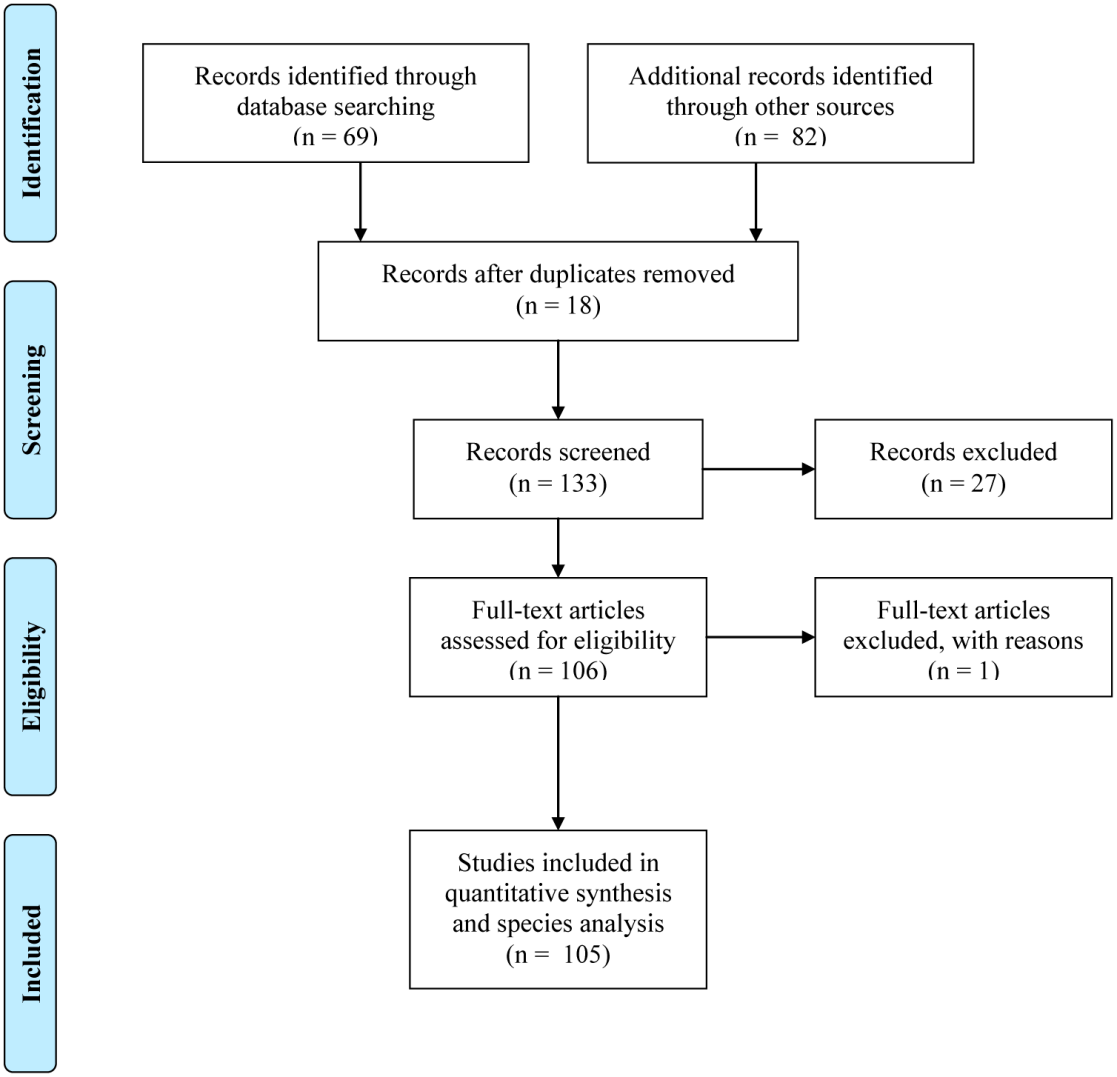
Flow chart using the Preferred Reporting Items for Systematic Reviews and Meta-Analyses statement.

To guarantee that only studies related to our research subject were included in our study, we first tracked all studies obtained from the combinations of keywords. Subsequently, we excluded studies that were not conducted in the Legal Brazilian Amazon and that did not involve the eight target species. The number of studies excluded and those retained were recorded for each of the screening stages according to the Preferred Reporting Items for Systematic Reviews and Meta-Analyses (PRISMA) statement (Fig 1) [33].

We examined, filtered, and collated the results returned by the searches to ensure that we considered all relevant studies that reported on the impact of subsistence hunting and the commercial use of the selected aquatic species. We then reviewed the relevant studies to extract the following data: 1) order of authorship, 2) authorship affiliation (including institution and country), 3) geographical location and coordinates of the study area, and 4) the species studied. If a document failed to provide geographical coordinates, we used Google Earth (GE) to obtain the approximate coordinates, supported by maps of the study area and key landmarks such as roads, rivers, islands, and other visual features that could be clearly distinguished in the GE images. All of the sites in our analyses were considered, even when studies reported more than one study site coordinate/location. We used ArcGIS 9.3 [34] to produce a final distribution map of all of the study sites that focused on the subsistence and commercial hunting of aquatic vertebrates within the Legal Brazilian Amazon.

### Compilation of research institutes/universities

From the results of the searches in the scientific and gray literature, we extracted the affiliations of the first and last authors in all of the documents. We chose the first and last authors as they traditionally represent those that most contributed intellectually to the paper. Usually the last author is also the senior and/or the head of the research group, and represents the senior investigator of the study. To better reflect collaboration networks in graduate theses, MSc theses, and PhD dissertations, we included the supervisor and co-supervisor (when clearly stated in the document) as co-authors. If the city for a university or institute was not cited in a document, then it was obtained from a Brazilian Ministry of Education database [35]. Coordinates of cities that contained Brazilian universities and research institutes were obtained from Instituto Brasileiro de Geografia e Estatística (IBGE, available at ftp://geoftp.ibge.gov.br/organizacao_territorial/localidades/), and coordinates of cities that contained international universities and research institutes were obtained using the information available from the website of the institute/university or GE. We used ArcGIS 9.3 [34] to produce a final distribution map of all the research institutions and universities of the first and last authors of the documents obtained by our literature survey. These points were overlaid with international and national borders obtained from Natural Earth (public domain, http://www.naturalearthdata.com/) and IBGE (public domain, http://downloads.ibge.gov.br/downloads_geociencias.htm). We considered the distance between research institutions and universities to be close when the linear distance between them was 500 km or less.

### Data analysis

Collaborations between research institutes/universities that published studies together were obtained and graphically represented with a spider diagram using the extension ET Geowizard 11.2 [36] for ArcGIS 9.3 [34]. We also used the package Igraph [37] in R [38] to graphically display collaboration networks among institutions.

We calculated the distances between the locations of the study sites and the locations of the research institutes/universities affiliated with the first and last authors of the same study in radians using the haversine formula [39] in R [38]. We then transformed the radian distances to kilometers. Finally, with the package Igraph [37] in R [38], we determined binary collaboration networks between national and international research institutes/universities and the aquatic vertebrate species. Binary network models are simple, and have been widely used in studies that have evaluated scientific collaboration networks [31, 40, 41]. We used the degree of node centrality [37] to characterize the network; the degree is the simplest of the node centrality measures, and only uses the local structure around nodes. In a binary network, the degree is the number of links possessed by a node [42].

## Results

### Spatial and temporal distribution of studies

**Fig 2:**
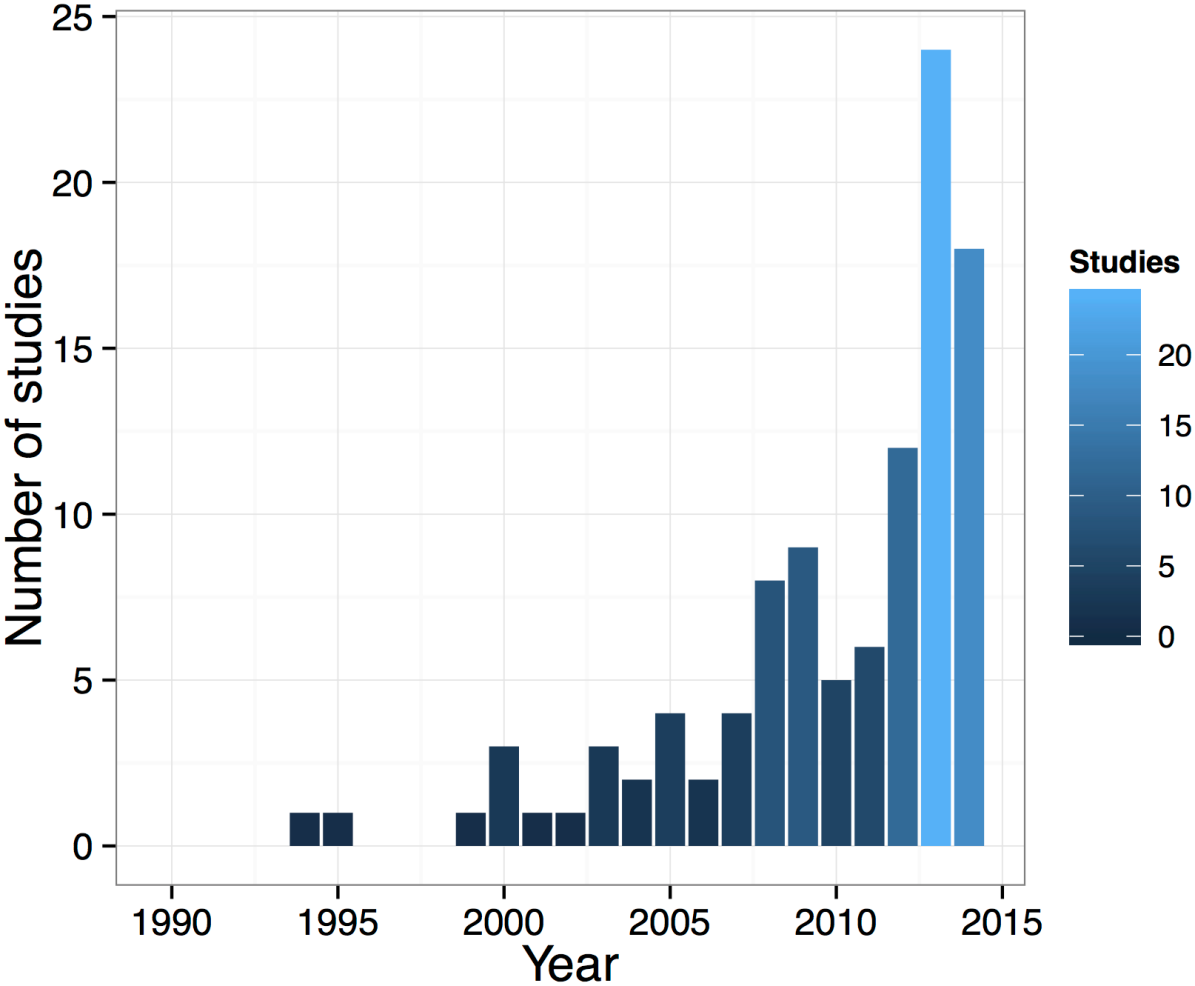
Annual number of studies of aquatic vertebrates from 1990 to 2014. The color gradient is proportional to the number of studies in each year.

Our searches returned 105 studies in 271 study locations in the Legal Brazilian Amazon in which the subsistence and/or commercial hunting of aquatic vertebrates occurred (S1 Table). Most studies were conducted after 2007 (80.6%). The highest number of studies was published in 2013 (n = 24), followed by 2014 (n = 18) (Fig 2).

**Fig 3:**
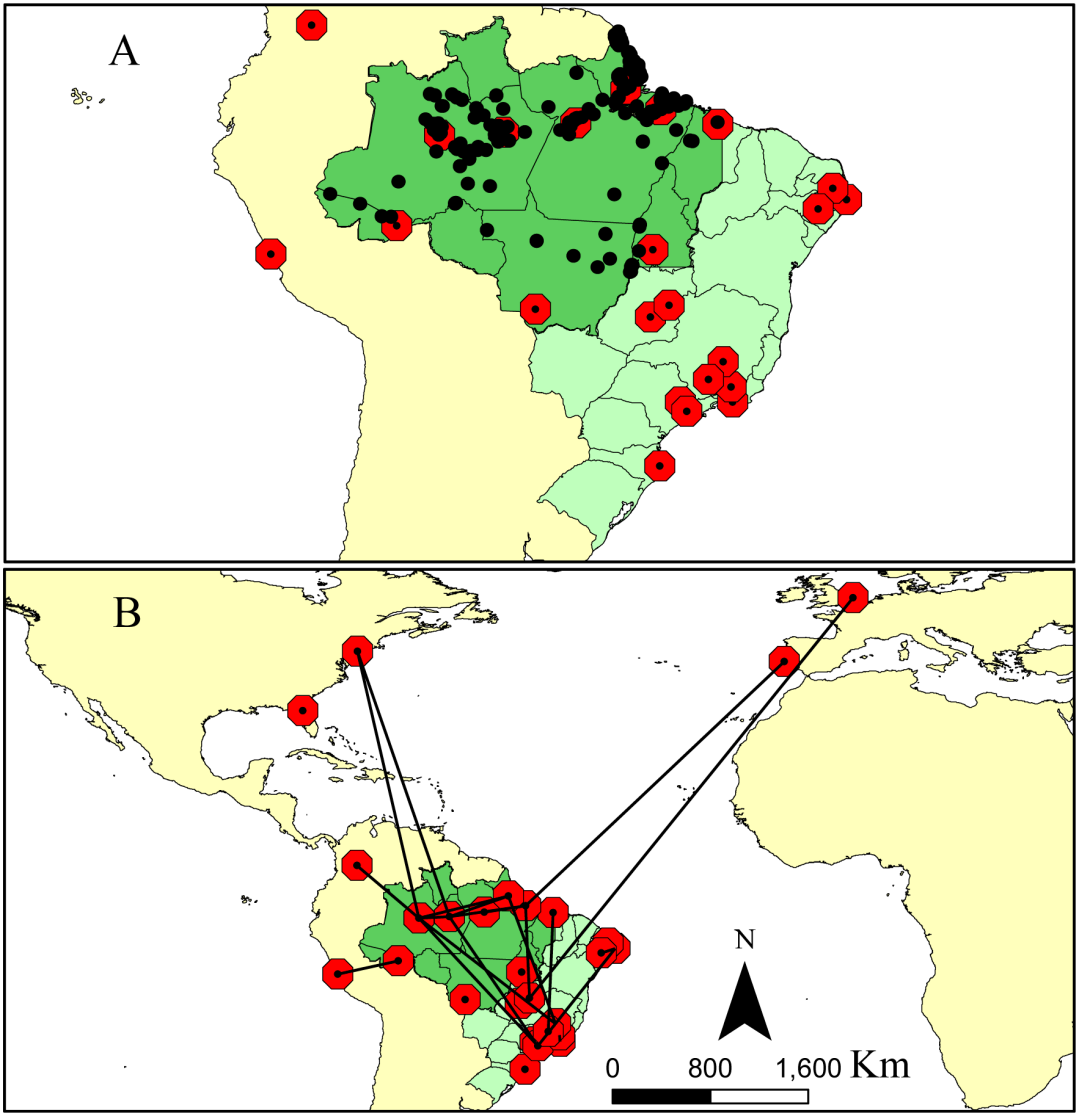
Locations of study regions in the Legal Brazilian Amazon. (A) Geographical distribution of studies with aquatic vertebrate species (black dots) and locations of research institutes (red circles with black dots) in South America; (B) Locations of national and international research institutes that performed the studies (red circles with black dots) and network collaborations among them (black lines).

The spatial distribution of the studies within the Legal Brazilian Amazon varied, but over 80% took place in the northeast and central Amazon and encompassed three States (Amazonas, 51.42%; Pará, 19.05%; and Amapá, 16.19%). The other regions of the Legal Brazilian Amazon had lower proportions of studies and involved another five States (Maranhão, 3.81%; Tocantins, 3.81%; Acre, 1.91%; Mato Grosso, 1.91%; Rondônia, 0.95%; and Roraima, 0.95%) (Fig 3).

### Collaboration networks of research institutes/universities on studies of aquatic vertebrates

**Fig 4:**
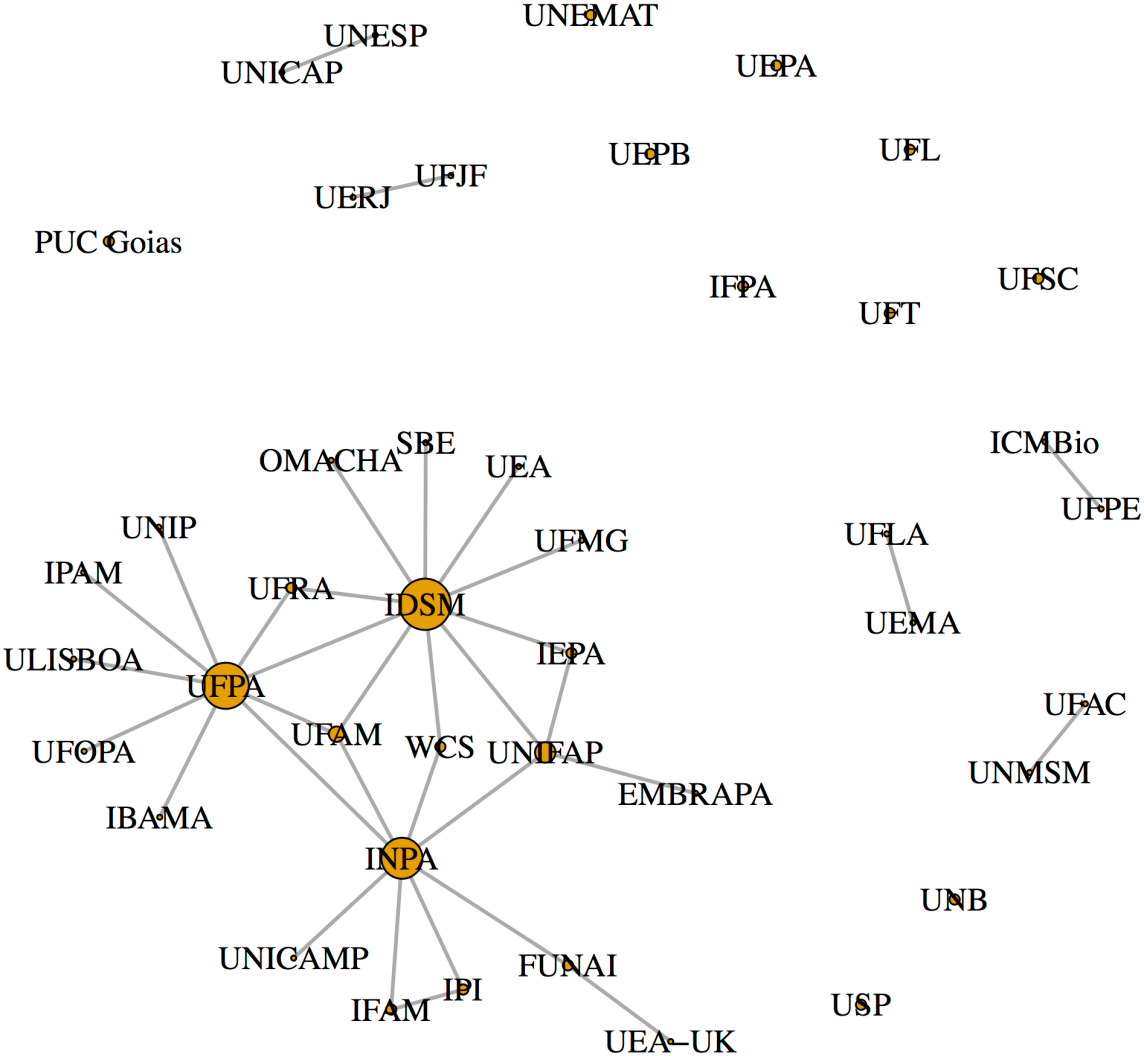
Scientific collaboration network on studies of aquatic Amazonian vertebrates. Each circle (node) represents an institution, and the lines connecting the nodes indicate collaborative relationships among institutions. Node sizes correspond to the degree of centrality.

The studies involved 43 institutions (37 Brazilian and 6 non-Brazilian) (Fig 3, S2 Table). Nearly half of the studies analyzed had up to two authors (n = 52, 49.52%), giving us confidence that our analysis using the first and last authors was representative of the dataset. Only 44.76% (n = 47) of the documents were scientific articles or book chapters; the majority were conference abstracts (n = 26) and graduate and MSc theses (n = 22), followed by PhD dissertations (n = 6), reports (n = 2), and magazine articles (n = 2). Some (n = 10, 23.26%) research institutes/universities did not collaborate with any other institute/university (Table 1). Considering only the 33 institutes/universities with collaborations, there were on average 2.06 collaborations per institution (range, 1–10, SD = 2.34). Three institutions had substantially more collaborations than the others, and represented the top 5% of the value range (IDSM, UFPA, and INPA, with 10, 9, and 8 collaborations, respectively; Table 1, Fig 4).

**Table 1:** List of institutions that were affiliated with the first and last authors of 105 studies of Amazonian aquatic species published from 1990 to 2014 and their respective collaborations with national and international institutions.

Institution^a^	Number of collaborative studies	Number of national collaborations	Number of international collaborations	National institution collaborations	International institution collaborations
EMBRAPA	1	1	0	UNIFAP	
FUNAI	2	1	1	INPA	UEA-UK
IBAMA	1	1	0	UFPA	
ICMBio	2	1	0	UFPE	
IDSM	10	8	2	SBE, UNIFAP, UFAM, UFMG, UFPA, UFRA, UEA, IEPA	WCS, OMACHA
IEPA	1	2	0	IDSM, UNIFAP	
IFAM	2	2	0	INPA, IPI	
IFPA	0	0	0		
INPA	9	7	1	UNICAMP, UFAM, UNIFAP, IPI, UFPA, IFAM, FUNAI	WCS
IPAM	1	1	0	UFPA	
IPI	3	2	0	INPA, IFAM	
OMACHA	1	0	1	IDSM	
SBE	1	1	0	IDSM	
PUC Goiás	0	0	0		
UEA	2	1	0	IDSM	
UEA-UK	1	0	1	FUNAI	
UEMA	1	1	0	UFLA	
UEPA	0	0	0		
UEPB	0	0	0		
UERJ	2	1	0	UFJF	
UFAC	1	0	1		UNMSM
UFAM	4	3	0	INPA, IDSM, UFPA	
UFJF	2	1	0	UERJ	
UFL	0	0	0		
UFLA	1	1	0	UEMA	
UFMG	1	1	0	IDSM	
UFOPA	1	1	0	UFPA	
UFPA	9	8	1	UFAM, UNIP, UFRA, IBAMA, UFOPA, INPA, IPAM, IDSM	ULISBOA
UFPE	2	1	0	ICMBio	
UFRA	1	2	0	UFPA, IDSM	
UFSC	0	0	0		
UFT	0	0	0		
ULISBOA	1	0	1	UFPA	
UNB	0	0	0		
UNEMAT	0	0	0		
UNESP	1	1	0	UNICAP	
UNICAMP	1	1	0	INPA	
UNICAP	1	1	0	UNESP	
UNIFAP	5	4	0	INPA, IDSM, EMBRAPA, IEPA	
UNIP	1	1	0	UFPA	
UNMSM	1	0	1	UFAC	
USP	0	0	0		
WCS	2	0	2	INPA, IDSM	

^a^ Institution full name (abbreviation): Empresa Brasileira de Pesquisa Agropecuária (EMBRAPA), Fundação Nacional do Índio (FUNAI), Instituto Brasileiro do Meio Ambiente e dos Recursos Naturais Renováveis (IBAMA), Instituto Chico Mendes de Conservação da Biodiversidade (ICMBio), Instituto de Desenvolvimento Sustentável Mamirauá (IDSM), Instituto de Pesquisa Cientifica e Tecnológica do Estado do Amapá (IEPA), Instituto Federal de Educação, Ciência e Tecnologia do Amazonas (IFAM), Instituto Federal do Pará (IFPA), Instituto Nacional de Pesquisas da Amazônia (INPA), Instituto de Pesquisa Ambiental da Amazônia (IPAM), Instituto Piagaçu (IPI), Fundación Omacha (OMACHA), Sociedade Brasileira de Espeleologia (SBE), Pontifícia Universidade Católica de Goiás (PUC Goiás), Universidade do Estado do Amazonas (UEA), University of East Anglia (UEA-UK), Universidade Estadual do Maranhão (UEMA), Universidade Estadual do Pará (UEPA), Universidade Estadual de Paraíba (UEPB), Universidade do Estado do Rio de Janeiro (UERJ), Universidade Federal do Acre (UFAC), Universidade Federal do Amazonas (UFAM), Universidade Federal de Juiz de Fora (UFJF), University of Florida (UFL), Universidade Federal de Lavras (UFLA), Universidade Federal de Minas Gerais (UFMG), Universidade Federal do Oeste do Pará (UFOPA), Universidade Federal do Pará (UFPA), Universidade Federal de Pernambuco (UFPE), Universidade Federal Rural da Amazônia (UFRA), Universidade Federal de Santa Catarina (UFSC), Universidade Federal do Tocantins (UFT), Universidade de Lisboa (ULISBOA), Universidade de Brasília (UNB), Universidade do Estado de Mato Grosso (UNEMAT), Universidade Estadual de São Paulo (UNESP), Universidade Estadual de Campinas (UNICAMP), Universidade Católica de Pernambuco (UNICAP), Universidade Federal do Amapá (UNIFAP), Universidade Paulista (UNIP), Universidad Nacional Mayor de San Marcos (UNMSM), Universidade de São Paulo (USP), Wildlife Conservation Society (WCS).

**Fig 5:**
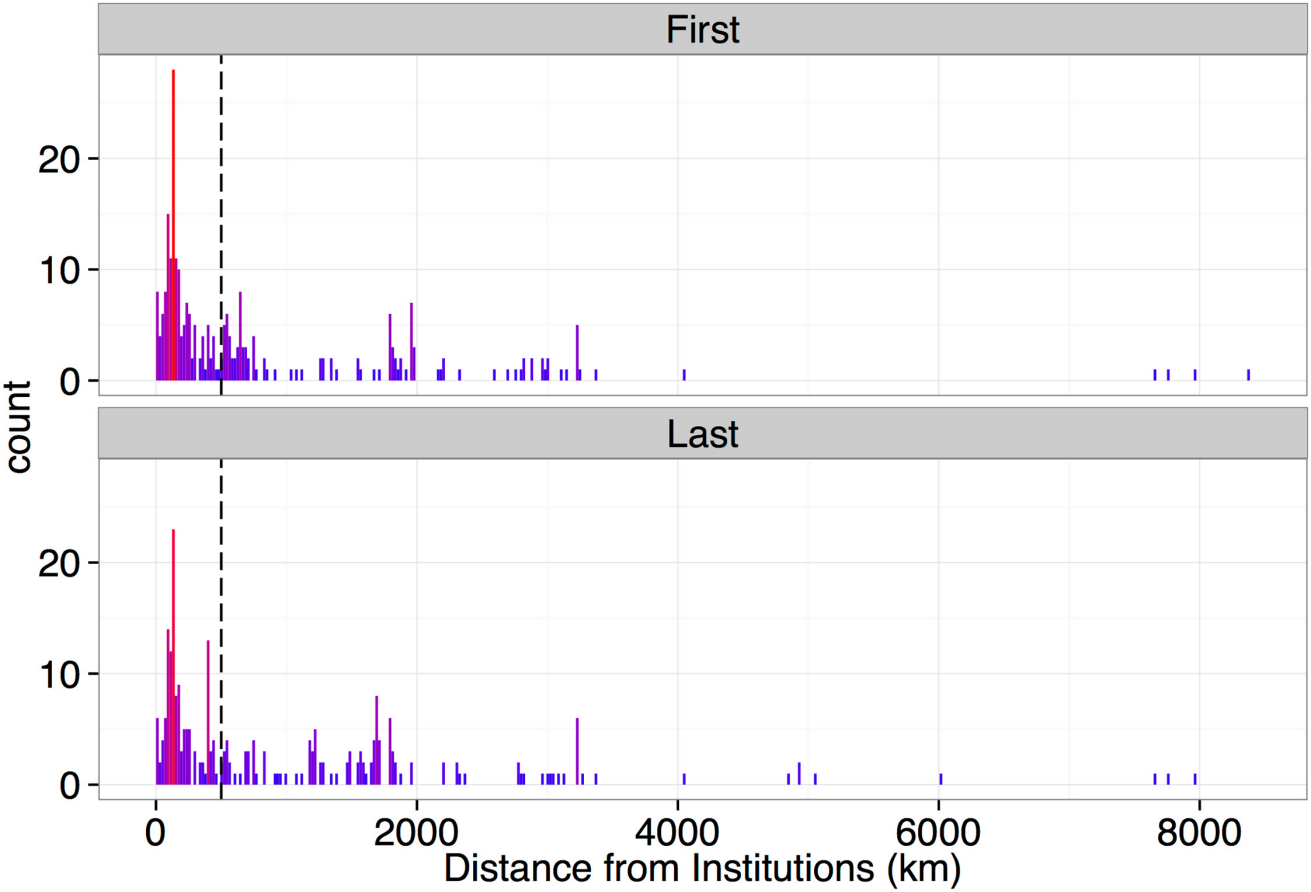
Distances (km) between locations of studies and locations of the first and last authors. The long dashed line shows the locations of studies conducted at 500-km distances from the authors’ affiliations. Red bars represent a high frequency of counts of distances from the institutions.

Overall, studies on the subsistence and/or commercial hunting of Amazonian aquatic vertebrates occurred more frequently in areas close to research institutes/universities: 52.9% of the research study sites were located within 500 km of the research institute/university of the first or last authors (Fig 5). There was a decrease in the number of studies when distances from the affiliations of the authors increased. International institutions (4000 km or more away) that conducted research on aquatic vertebrates were exceptions to this trend.

**Fig 6:**
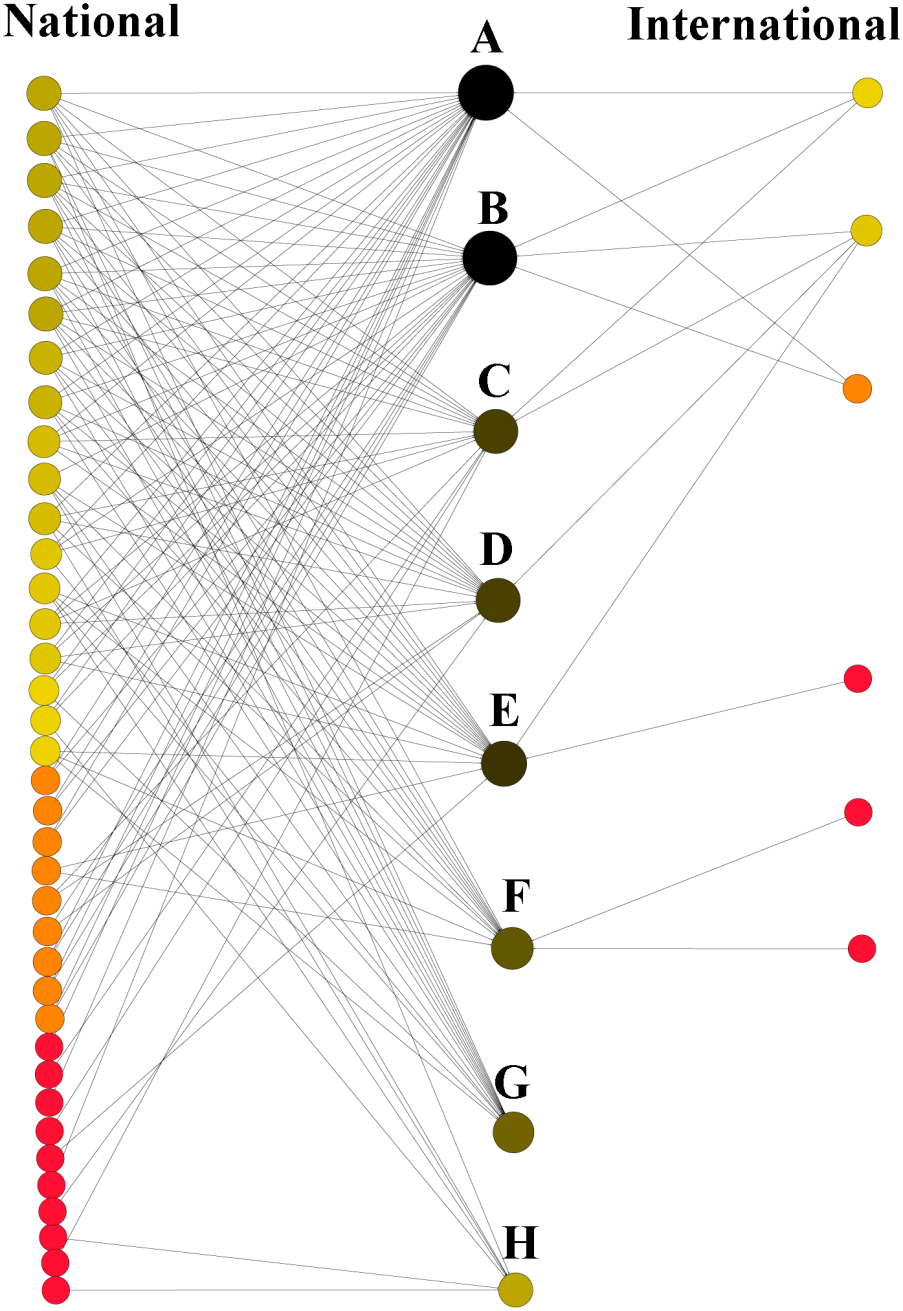
Contribution of each national and international research institute/university in the scientific collaboration network. Sizes and colors of the circles represent the contribution of each institute to the network. Aquatic species (A–H) are shown in descending order of number of studies in the Legal Brazilian Amazon. A, *Podocnemis unifilis*; B, *Podocnemis expansa*; C, *Caiman crocodilus*; D, *Melanosuchus niger*; E, *Inia geoffrensis*; F, *Trichechus inunguis*; G, *Sotalia fluviatilis*; H, *Trichechus manatus*.

Some research institutes/universities, such as IDSM, INPA, IPI, UFPA, UFRA, and UNESP, conducted many studies and had large collaboration networks, and contributed most to the network of studies on Amazonian aquatic vertebrates (Table 1, Fig 6). Among the species selected, the most studied was the yellow-spotted river turtle, followed by the South American river turtle, the common caiman, the black caiman, the Boto, the South American manatee, the Tucuxi, and the West Indian manatee (Fig 6). The same trend was observed in contributions to the network structure in degrees: a large number of institutions were involved in studies of the two species of freshwater turtle.

Only one institution (IDSM) in the collaboration network performed studies on all of the aquatic species evaluated, while other institutions (INPA, UFPA, UFRA, UNESP, and UNIFAP) performed studies on seven species (Table 2). The majority of the institutions (IBAMA, ICMBio, IFPA, OMACHA, UEMA, UFLA, UFPE, UNB, UNEMAT, UFL, and ULISBOA) studied only one aquatic species (Table 2).

**Table 2:** List of Amazonian aquatic species obtained from 105 studies published from 1990 to 2014 that were studied by each institution, institution type, and country, and contributions of species-institution combinations to the scientific collaboration network measured in normalized degrees.

Institution^a^	Institution type	Country	Contribution to network structure in normalized degrees							
			South American river turtle	Yellow-spotted river turtle	Black caiman	Common caiman	South American manatee	West Indian manatee	Tucuxi	Boto
EMBRAPA	Research institution	Brazil	0.00	0.02	0.00	0.00	0.00	0.00	0.00	0.00
FUNAI	Foundation	Brazil	0.02	0.00	0.04	0.00	0.00	0.00	0.00	0.00
IBAMA	Research institution	Brazil	0.00	0.00	0.04	0.00	0.00	0.00	0.00	0.00
ICMBio	Research institution	Brazil	0.00	0.00	0.00	0.00	0.00	0.11	0.00	0.00
IDSM	Research institution	Brazil	0.02	0.06	0.11	0.13	0.22	0.11	0.20	0.14
IEPA	Research institution	Brazil	0.00	0.00	0.00	0.00	0.04	0.11	0.04	0.03
IFAM	University	Brazil	0.05	0.04	0.00	0.00	0.00	0.00	0.00	0.00
IFPA	University	Brazil	0.00	0.00	0.00	0.00	0.00	0.00	0.00	0.03
INPA	Research institution	Brazil	0.14	0.14	0.11	0.13	0.13	0.00	0.04	0.14
IPAM	Research institution	Brazil	0.02	0.02	0.04	0.04	0.00	0.00	0.00	0.00
IPI	Research institution	Brazil	0.05	0.04	0.04	0.04	0.04	0.00	0.04	0.03
OMACHA	Non-governmental organization	Colombia	0.00	0.00	0.00	0.00	0.00	0.00	0.04	0.00
SBE	Association	Brazil	0.00	0.02	0.04	0.04	0.00	0.11	0.04	0.00
PUC Goiás	University	Brazil	0.00	0.00	0.04	0.04	0.00	0.00	0.00	0.00
UEA	University	Brazil	0.05	0.04	0.00	0.00	0.00	0.11	0.04	0.03
UEMA	University	Brazil	0.00	0.00	0.00	0.04	0.00	0.00	0.00	0.00
UEPA	University	Brazil	0.00	0.00	0.00	0.00	0.04	0.00	0.04	0.03
UEPB	University	Brazil	0.02	0.00	0.00	0.00	0.04	0.11	0.08	0.07
UERJ	University	Brazil	0.02	0.02	0.04	0.00	0.04	0.00	0.04	0.03
UFAC	University	Brazil	0.02	0.02	0.00	0.00	0.00	0.00	0.00	0.00
UFAM	University	Brazil	0.02	0.06	0.04	0.08	0.00	0.00	0.00	0.00
UFJF	University	Brazil	0.02	0.04	0.04	0.00	0.04	0.00	0.04	0.03
UFLA	University	Brazil	0.00	0.00	0.00	0.04	0.00	0.00	0.00	0.00
UFMG	University	Brazil	0.02	0.02	0.04	0.00	0.00	0.00	0.00	0.07
UFOPA	University	Brazil	0.02	0.02	0.00	0.00	0.00	0.00	0.00	0.00
UFPA	University	Brazil	0.09	0.10	0.22	0.13	0.13	0.00	0.16	0.10
UFPE	University	Brazil	0.00	0.00	0.00	0.00	0.00	0.11	0.00	0.00
UFRA	University	Brazil	0.05	0.04	0.11	0.08	0.09	0.00	0.04	0.03
UFSC	University	Brazil	0.00	0.00	0.00	0.00	0.00	0.00	0.04	0.03
UFT	University	Brazil	0.05	0.04	0.00	0.00	0.00	0.00	0.00	0.00
UNB	University	Brazil	0.00	0.02	0.00	0.00	0.00	0.00	0.00	0.00
UNEMAT	University	Brazil	0.00	0.02	0.00	0.00	0.00	0.00	0.00	0.00
UNESP	University	Brazil	0.05	0.02	0.04	0.04	0.04	0.11	0.00	0.03
UNICAMP	University	Brazil	0.05	0.04	0.00	0.00	0.00	0.00	0.00	0.00
UNICAP	University	Brazil	0.02	0.02	0.00	0.00	0.04	0.00	0.00	0.00
UNIFAP	University	Brazil	0.07	0.04	0.00	0.04	0.09	0.11	0.08	0.07
UNIP	University	Brazil	0.02	0.02	0.00	0.00	0.00	0.00	0.00	0.00
USP	University	Brazil	0.00	0.00	0.00	0.04	0.00	0.00	0.00	0.00
UFL	University	United States	0.00	0.00	0.00	0.00	0.00	0.00	0.00	0.03
UNMSM	University	Peru	0.02	0.02	0.00	0.00	0.00	0.00	0.00	0.00
ULISBOA	University	Portugal	0.00	0.00	0.00	0.00	0.00	0.00	0.04	0.00
UEA-UK	University	England	0.05	0.02	0.00	0.04	0.00	0.00	0.00	0.00
WCS	Non-governmental organization	United States	0.02	0.02	0.04	0.04	0.00	0.00	0.00	0.03

^a^ Institution full name (abbreviation): Empresa Brasileira de Pesquisa Agropecuária (EMBRAPA), Fundação Nacional do Índio (FUNAI), Instituto Brasileiro do Meio Ambiente e dos Recursos Naturais Renováveis (IBAMA), Instituto Chico Mendes de Conservação da Biodiversidade (ICMBio), Instituto de Desenvolvimento Sustentável Mamirauá (IDSM), Instituto de Pesquisa Cientifica e Tecnológica do Estado do Amapá (IEPA), Instituto Federal de Educação, Ciência e Tecnologia do Amazonas (IFAM), Instituto Federal do Pará (IFPA), Instituto Nacional de Pesquisas da Amazônia (INPA), Instituto de Pesquisa Ambiental da Amazônia (IPAM), Instituto Piagaçu (IPI), Fundación Omacha (OMACHA), Sociedade Brasileira de Espeleologia (SBE), Pontifícia Universidade Católica de Goiás (PUC Goiás), Universidade do Estado do Amazonas (UEA), Universidade Estadual do Maranhão (UEMA), Universidade Estadual do Pará (UEPA), Universidade Estadual de Paraíba (UEPB), Universidade do Estado do Rio de Janeiro (UERJ), Universidade Federal do Acre (UFAC), Universidade Federal do Amazonas (UFAM), Universidade Federal de Juiz de Fora (UFJF), Universidade Federal de Lavras (UFLA), Universidade Federal de Minas Gerais (UFMG), Universidade Federal do Oeste do Pará (UFOPA), Universidade Federal do Pará (UFPA), Universidade Federal de Pernambuco (UFPE), Universidade Federal Rural da Amazônia (UFRA), Universidade Federal de Santa Catarina (UFSC), Universidade Federal do Tocantins (UFT), Universidade de Brasília (UNB), Universidade do Estado de Mato Grosso (UNEMAT), Universidade Estadual de São Paulo (UNESP), Universidade Estadual de Campinas (UNICAMP), Universidade Católica de Pernambuco (UNICAP), Universidade Federal do Amapá (UNIFAP), Universidade Paulista (UNIP), Universidade de São Paulo (USP), University of Florida (UFL), Universidad Nacional Mayor de San Marcos (UNMSM), Universidade de Lisboa (ULISBOA), University of East Anglia (UEA-UK), Wildlife Conservation Society (WCS).

## Discussion

In recent years, there has been an increase in the number of studies on Amazonian aquatic vertebrates and most of them have been multi-authored, indicating that scientific collaboration plays an important role in the study of aquatic vertebrates. Although international and interdisciplinary scientific collaboration has expanded rapidly in all fields of research [43] and increases citation rates of ecological articles [44], we found that there have been few international collaborations on Amazonian aquatic vertebrates over the past 24 years. Consequently, there is a huge opportunity for national and international research institutes to collaborate in the study of aquatic vertebrates in the largest remaining expanse of tropical forest in the world.

Of the species evaluated, the two species of freshwater turtle (*P*. *unifilis* and *P*. *expansa*) were the most studied. Freshwater turtles have a long history of consumption by Brazilian Amazon riverine communities [9, 45], and this has generated great attention from the scientific community. As protein acquisition for local communities in tropical regions relies heavily on meat from wild vertebrates [4, 5], it is expected that this subject will attract research interest. Studies that have investigated human exploitation effects on aquatic vertebrates have increased in the past 10 years.

However, some aquatic species, such as manatees and Botos, are still poorly studied. This could be related to the difficulty of studying these animals in the wild, because of their biology and natural history [11, 12]. The semi-aquatic nature of freshwater turtles, including their habits of basking and beach-nesting [10], makes them easier subjects to study than the other species included here. Caimans also have the habit of basking on fallen trees and in open areas close to riverbanks, and care for their eggs and hatchlings on riverbanks [46]. Therefore, the fact that caimans are the most studied species after freshwater turtles was to be expected. There is a myriad of uses of aquatic species in the Brazilian Amazon that enhances their potential interest for scientific research. These uses include traditional medicine [13] and religious beliefs [14, 47], which interact additively or synergistically with meat consumption and make the study of these species challenging.

Scientific collaborations have been developing interdisciplinary approaches in recent years [48]. Overall, 43 research institutes were included in our 105 studies, 33 of which had some collaboration with other institutes. Institutes such as IDSM, UFPA, and INPA had the highest number of collaborations. In Amazonia, these institutes are known to have high international connectivity due to the number of international researchers hired in each institute, and to their long history of studies on ecology. INPA and UFPA were created in 1952 and 1957, respectively, and are amongst the most traditional research institutes in the Brazilian Amazon. In contrast, some institutes did not have any collaborations, and worked alone in their studies on aquatic vertebrates (IFPA, PUC Goiás, UEPA, UEPB, UFL, UFSC, UFT, UNB, UNEMAT, and USP). A potential limitation of our study was that we analyzed documents only using the first and last authors; datasets including all authors from all research groups would provide a better overview of the scientific network of collaborations.

Our study demonstrates that the locations of aquatic vertebrate study sites were close to the research institutes of the first and last authors. Over 50% of the studies were conducted within 500 km of the institute to which the researchers were affiliated to, so the spatial distribution of studies on Amazonian aquatic vertebrates has a clear bias that needs to be better explored and analyzed.

Faculty effort and time commitments for developing and delivering graduate courses have been evaluated previously [49]; however, to our knowledge, no studies have been conducted that have evaluated the effort and time researchers allocate in order to conduct field research in ecology. There are obvious constraints in terms of costs and time if researchers need to travel long distances to conduct fieldwork. However, in studies on subsistence and commercial hunting, there are increasing levels of hunting in areas close to human settlements [4]. Therefore, our study provides clear evidence that the development of new strategies and improvements in scientific collaboration [44, 48] will benefit studies on aquatic vertebrates.

An enhanced understanding of the dynamics of scientific collaborations will help to direct efforts and improve communication effectiveness, enhance interactions between institutes, and reduce costs from a teamwork perspective [50].

## Conclusions

Studies on Amazonian aquatic vertebrates will become increasingly important for tropical wildlife conservation because of the growing number of built and planned hydroelectric dams in the Amazon region. In the present study, we have shown that most of the studies that have been conducted were within 500 km of the lead authors’ affiliated institutes. This indicates a strong potential bias in studies of the subsistence and commercial hunting of aquatic vertebrates. We also show that improvements in national, and particularly international, collaborations would benefit both the studies and the research institutes by increasing article citation rates and reducing the costs associated with fieldwork. To enhance scientific collaborations in studies on Amazonian aquatic vertebrates, we consider it essential to improve communication between research institutes and researchers and increase the distance between survey sites and the institutes to which the researchers are affiliated.

## Supporting Information

S1 Table List of 105 studies on the subsistence and/or commercial hunting of Amazonian aquatic vertebrates. (DOC)

S2 Table List of research institutes/universities that conducted studies on Amazonian aquatic vertebrates. (DOC)
