# Supplementary material for: Implications of Scientific Collaboration Networks on Studies of Aquatic Vertebrates in the Brazilian Amazon
Source: PLoS One. 2016 Jun 28;11(6):e0158413. doi: 10.1371/journal.pone.0158413 (PMC4924867; doi:10.1371/journal.pone.0158413)
Supplement: S1 Table — (DOC) [file pone.0158413.s001.doc]

**S1 Table. List of 105 studies on the subsistence and/or commercial hunting of Amazonian aquatic vertebrates.**

List of 105 studies and 271 locations of sites where research on subsistence and/or commercial hunting of Amazonian aquatic vertebrates from 1990 to 2014 was conducted.

| **Source** | **Type of literature** | **Source**  **data** | **Map** | **Coordinates** |
| --- | --- | --- | --- | --- |
| [1] | Graduate thesis | Other | No | 0°52'19.18''N;  50°02'44.67''W |
|  | Graduate thesis | Other | No | 0°54'21.19''N;  50°01'04.99''W |
| [2] | MSc thesis | Google | Yes | 1°04'22.05''N;  51°30'20.60''W |
|  | MSc thesis | Google | Yes | 1°03'33.99''N;  51°31'29.86''W |
|  | MSc thesis | Google | Yes | 1°03'26.44''N;  51°31'24.13''W |
|  | MSc thesis | Google | Yes | 1°02'34.57''N;  51°31'51.09''W |
|  | MSc thesis | Google | Yes | 1°00'59.63''N;  51°35'40.10''W |
|  | MSc thesis | Google | Yes | 1°00'25.63''N;  51°35'50.25''W |
|  | MSc thesis | Google | Yes | 0°59'33.62''N;  51°36'00.00''W |
|  | MSc thesis | Google | Yes | 0°58'47.40''N;  51°35'04.08''W |
|  | MSc thesis | Google | Yes | 0°58'29.03''N;  51°35'18.29''W |
|  | MSc thesis | Google | Yes | 0°57'13.53''N;  51°35'59.45''W |
|  | MSc thesis | Google | Yes | 0º55'58.96''N; 51º35'43.93''W |
|  | MSc thesis | Google | Yes | 0º44'32.15''N; 51º28'35.99''W |
|  | MSc thesis | Google | Yes | 0º44'31.82''N; 51º27'25.61''W |
|  | MSc thesis | Google | Yes | 0º44'20.61''N; 51º27'23.02''W |
|  | MSc thesis | Google | Yes | 0º44'10.60''N; 51º27'20.28''W |
|  | MSc thesis | Google | Yes | 0º43'51.85''N; 51º27'26.94''W |
|  | MSc thesis | Google | Yes | 0º42'40.92''N; 51º26'07.11''W |
|  | MSc thesis | Google | Yes | 0º43'11.94''N; 51º24'53.26''W |
|  | MSc thesis | Google | Yes | 0º44'03.45''N; 51º23'28.44''W |
|  | MSc thesis | Google | Yes | 0º44'31.99''N; 51º22'29.91''W |
|  | MSc thesis | Google | Yes | 0º44'39.25''N; 51º22'21.17''W |
| [3] | MSc thesis | Google | Yes | 3º12'53.27''N; 51º32'59.56''W |
|  | MSc thesis | Google | Yes | 3º20'35.83''N; 51º39'18.49''W |
|  | MSc thesis | Google | Yes | 3º22'46.81''N; 51º41'33.74''W |
|  | MSc thesis | Google | Yes | 3º30'11.95''N; 51º44'41.29''W |
|  | MSc thesis | Google | Yes | 3º36'6.09''N; 51º42'27.80''W |
|  | MSc thesis | Google | Yes | 3º44'00.22''N; 51º43'32.89''W |
|  | MSc thesis | Google | Yes | 3º23'17.49''N; 51º17'00.83''W |
|  | MSc thesis | Google | Yes | 3º27'21.87''N; 51º31'41.33''W |
|  | MSc thesis | Google | Yes | 3º35'21.70''N; 51º23'59.16''W |
|  | MSc thesis | Google | Yes | 3º39'39.37''N; 51º22'32.70''W |
|  | MSc thesis | Google | Yes | 3º46'1.78''N; 51º35'26.81''W |
|  | MSc thesis | Google | Yes | 3º53'50.34''N; 51º28'59.68''W |
| [4] | Scientific literature | ISI | No | 0°55'29"N; 51°35'45"W |
| [5] | Graduate thesis | Other | Yes | 4º15'22.77''N; 51º36'08.07''W |
|  | Graduate thesis | Other | Yes | 2º44'17.1''N; 50º47'20.45''W |
|  | Graduate thesis | Other | Yes | 2º40'57.42''N; 50º50'52.41''W |
|  | Graduate thesis | Other | Yes | 2º29'36.37''N; 50º50'05.32''W |
|  | Graduate thesis | Other | Yes | 2º06'50.78''N; 50º25'06.66''W |
|  | Graduate thesis | Other | Yes | 2º08'43.44''N; 50º41'21.3''W |
|  | Graduate thesis | Other | Yes | 2º04'56.66''N; 50º36'04.31''W |
|  | Graduate thesis | Other | Yes | 2º03'07.07''N; 50º45'29.54''W |
|  | Graduate thesis | Other | Yes | 1º48'18.84''N; 50º25'46.70''W |
|  | Graduate thesis | Other | Yes | 1º41'18.36''N; 50º44'13.53''W |
|  | Graduate thesis | Other | Yes | 1º25'35.90''N; 50º35'04.25''W |
|  | Graduate thesis | Other | Yes | 1º19'28.50''N; 50º08'11.16''W |
|  | Graduate thesis | Other | Yes | 0º59'06.90''N; 49º53'57.52''W |
|  | Graduate thesis | Other | Yes | 0º57'07.96''N; 49º57'16.93''W |
|  | Graduate thesis | Other | Yes | 0º55'43.18''N; 49º59'39.42''W |
|  | Graduate thesis | Other | Yes | 0º54'40.07''N; 49º57'11.78''W |
|  | Graduate thesis | Other | Yes | 0º53'41.33''N; 50º17'14.15''W |
|  | Graduate thesis | Other | Yes | 0º44'02.66''N; 50º08'43.89''W |
|  | Graduate thesis | Other | Yes | 0º13'46.81''N; 50º45'11.77''W |
|  | Graduate thesis | Other | Yes | 0º16'29.73''N; 50º48'39.54''W |
|  | Graduate thesis | Other | Yes | 0º08'57.61''N; 50º56'01.96''W |
|  | Graduate thesis | Other | Yes | 0º03'22.98''S; 51º05'39.05''W |
|  | Graduate thesis | Other | Yes | 0º04'55.54''S; 51º05'09.35''W |
|  | Graduate thesis | Other | Yes | 0º33'20.73''S; 51º33'04.41''W |
|  | Graduate thesis | Other | Yes | 0º32'08.41''S; 51º35'48.57''W |
|  | Graduate thesis | Other | Yes | 0º33'17.97''S; 51º35'11.54''W |
|  | Graduate thesis | Other | Yes | 0º47'56.42''S; 51º42'41.49''W |
|  | Graduate thesis | Other | Yes | 0º48'45.90''S; 51º43'28.65''W |
| [6] | PhD dissertation | Other | No | 0º04'28.24''S; 51º10'24.54''W |
| [7] | Report | Google | No | 1º20'29.1''N; 50º13'32.18''W |
| [8] | Report | Other | No | 1º14'41.03''N; 49º59'29.44''W |
|  | Report | Other | No | 1º41'5.86''N;  50º6'49.27''W |
|  | Report | Other | No | 1º40'38.15''N;  50º10'25.47''W |
|  | Report | Other | No | 1°48'25"N; 50°7'36.2"W |
|  | Report | Other | No | 1°40'4.9"N; 50°7'2.9"W |
|  | Report | Other | No | 1°42'4.2"N; 50°08'38.9"W |
|  | Report | Other | No | 1°37'58.2"N; 50°9'43.1"W |
|  | Report | Other | No | 1°42'18.6"N; 50°12'58.7"W |
|  | Report | Other | No | 1°44'19.4''N; 50°12'21''W |
|  | Report | Other | No | 1°45'28.3"N; 50°14'40"W |
|  | Report | Other | No | 1°45'57.5"N; 50°17'28.1"W |
| [9] | Graduate thesis | Other | Yes | 1º6' 32.04''N;  51º27'47.89''W |
|  | Graduate thesis | Other | Yes | 1º6'27.51''N;  51º27'26.72''W |
|  | Graduate thesis | Other | Yes | 1º6'22.98''N;  51º27'56.37''W |
|  | Graduate thesis | Other | Yes | 1º5'52.95''N;  51º28'34.22''W |
|  | Graduate thesis | Other | Yes | 1º5'12.68''N;  51º29'32.26''W |
|  | Graduate thesis | Other | Yes | 1º5'4.87''N;  51º29'18.70''W |
|  | Graduate thesis | Other | Yes | 1º4'47.83''N;  51º30'22.38''W |
|  | Graduate thesis | Other | Yes | 1º4'27.92''N;  51º30'20.17''W |
|  | Graduate thesis | Other | Yes | 1º4'26.52''N;  5º30'20.43''W |
|  | Graduate thesis | Other | Yes | 1º3'33.01''N;  51º31'29.41''W |
|  | Graduate thesis | Other | Yes | 1º2'56.41''N;  51º32'50.48''W |
|  | Graduate thesis | Other | Yes | 1º2'51.39''N;  51º33'40.09''W |
|  | Graduate thesis | Other | Yes | 1º2'38.13''N;  51º33'46.95''W |
|  | Graduate thesis | Other | Yes | 1º1'4.54''N;  51º35'40.79''W |
|  | Graduate thesis | Other | Yes | 1º1'4.48''N;  51º35'28.72''W |
|  | Graduate thesis | Other | Yes | 1º0' 36.89''N;  51º35'45.28''W |
|  | Graduate thesis | Other | Yes | 1º0'24.97''N;  51º35'52.14''W |
|  | Graduate thesis | Other | Yes | 1º0'18.29''N;  51º35'53.49''W |
|  | Graduate thesis | Other | Yes | 0º58'46.46''N;  51º35'4.59''W |
|  | Graduate thesis | Other | Yes | 0º57'4.25''N; 51º35'58.89''W |
| [10] | Graduate thesis | Other | Yes | 1º19'14.2''N; 50º16'54.40''W |
|  | Graduate thesis | Other | Yes | 1º14'5.98''N; 50º16'34.28''W |
| [11] | Scientific literature | Google | No | 0º53'02.80''N; 50º07'59.28''W |
|  | Scientific literature | Google | No | 3º50'26.69''N; 51º50'01.16''W |
| [12] | Conference abstract | Other | No | 1°05'10"S;  51°46'36"W |
| [13] | Scientific literature | Google | No | 2º4'59.99''S;  62º4'59.99''W |
| [14] | Scientific literature | Google | No | 4°26’S; 61°17'W |
|  | Scientific literature | Google | No | 4°13'S; 61°51'W |
|  | Scientific literature | Google | No | 4°16'S; 61°51'W |
| [15] | Scientific literature | ISI | No | 2º42'06.56''S; 65º06'08.12''W |
| [16] | Scientific literature | ISI | No | 2º42'06.56''S; 65º06'08.12''W |
| [17] | MSc thesis | Google | No | 4º24'20.3''S; 61º54'07.07''W |
| [18] | Scientific literature | ISI | No | 2º10'44''S;  65º42'27.06''W |
| [19] | Scientific literature | ISI | No | 3°18'10"S; 60°37'18"W |
| [20] | Graduate thesis | Other | No | 12°51'40.57''S; 50°35'33.75''W |
|  | Graduate thesis | Other | No | 12°52'5.77''S; 50°33'8.41''W |
|  | Graduate thesis | Other | No | 13° 21' 53.84''S; 50°39'37.58''W |
|  | Graduate thesis | Other | No | 13°4'46.11''S; 50°37'59.08''W |
|  | Graduate thesis | Other | No | 12°51'40.57''S;  50°35'33.75''W |
|  | Graduate thesis | Other | No | 13°2'38.12''S  50°37'33.83''W |
|  | Graduate thesis | Other | No | 13°0'8.79''S  50°39'24.44''W |
|  | Graduate thesis | Other | No | 13°24'17.84''S  50°43'45.34''W |
|  | Graduate thesis | Other | No | 8°19'1.41''S;  63°29'1.58''W |
|  | Graduate thesis | Other | No | 8°18'53.84''S;  63°29'47.47''W |
|  | Graduate thesis | Other | No | 8°20'9.80''S;  63°29'25.50''W |
|  | Graduate thesis | Other | No | 8°21'44.35''S;  63°32'24.66''W |
|  | Graduate thesis | Other | No | 8°21'37.84''S;  63°32'24.59''W |
|  | Graduate thesis | Other | No | 8°22'38.72''S;  63°33'27.87''W |
| [21] | Scientific literature | Google | No | 10º01'25.90''S; 50º01'27.07''W |
| [22] | Scientific literature | Google | No | 4°00'N; 51°50'W |
| [23] | Scientific literature | Google | No | 2º29'07''S; 64º44'53''W |
|  | Scientific literature | Google | No | 2º44'37''S; 64º29'51''W |
|  | Scientific literature | Google | No | 2º49'06''S; 64º37'14''W |
| [24] | Chapter | Other | No | 2°51'S; 64°54'W |
|  | Scientific literature | Other | No | 2º53'S; 64º51'W |
| [25] | Chapter | Google | No | 3º01'42.38''S; 60º15'56.04''W |
| [26] | Scientific literature | ISI | Yes | 2º28'37''S; 65º20'04''W |
|  | Scientific literature | ISI | Yes | 2º47'52''S; 65º0'57''W |
|  | Scientific literature | ISI | Yes | 2º56'41''S; 65º04'30''W |
|  | Scientific literature | ISI | Yes | 3º06'59''S; 64º47'36''W |
| [27] | Conference abstract | Other | No | 2º10'44''S; 65º42'27.06''W |
| [28] | MSc thesis | Google | Yes | 2º44'46.80''S; 60º26'11.18''W |
|  | MSc thesis | Google | Yes | 2º43'21.08''S; 60º25'02.49''W |
|  | MSc thesis | Google | Yes | 2º47'07.93''S; 60º26'57.35''W |
|  | MSc thesis | Google | Yes | 2º48'52.43''S; 60º28'51.32''W |
|  | MSc thesis | Google | Yes | 2º40'09.40''S; 60º19'10.49''W |
| [29] | Scientific literature | Google | No | 3º18'15"S; 60º37'03"W |
| [30] | MSc thesis | Other | Yes | 12º10'31.43''S; 64º27'04.97''W |
|  | MSc thesis | Other | Yes | 12º11'05.81''S; 64º27'50.29''W |
|  | MSc thesis | Other | Yes | 12º12'19.67''S. 64º29'01.61''W |
|  | MSc thesis | Other | Yes | 12º13'29.91''S; 64º31'05.61''W |
|  | MSc thesis | Other | Yes | 12º11'04.30''S; 64º27'44.33''W |
| [31] | Magazine | Google | No | 2º10'44''S; 65º42'27.06''W |
| [32] | Scientific literature | ISI | No | 5°37'S; 63º11'W |
| [33] | Scientific literature | Google | No | 9º17'07.54''S; 68º53'32.65''W |
|  | Scientific literature | Google | No | 8º23'22.10''S; 70º29'47.72''W |
|  | Scientific literature | Google | No | 7º39'35.61''S; 72º40'19.11''W |
| [34] | Scientific literature | ISI | No | 1º27'21''S; 48°30'16''W |
| [35] | Scientific literature | ISI | No | 2º24'29.00''S; 44º19'22.76''W |
| [36] | Scientific literature | ISI | No | 1º27'21''S; 48°30'16''W |
|  | Scientific literature | ISI | No | 0º40'09.88''S; 48º28'55.75''W |
| [37] | MSc thesis | Google | No | 2º58'S; 55º50'W |
|  | MSc thesis | Google | No | 2º17'37.77''S; 54º49'50.67''W |
|  | MSc thesis | Google | No | 2º23'27.14''S; 55º12'02.07''W |
|  | MSc thesis | Google | No | 2º42'18.20''S; 55º04'57.28''W |
| [38] | Scientific literature | ISI | No | 0º19'45.38''S; 65º22'22.84''W |
| [39] | Scientific literature | ISI | Yes | 0°44'59.66"S; 52°43'13.19"W |
| [40] | MSc thesis | Other | Yes | 12º04'14.14''S; 50º12'16.31''W |
|  | MSc thesis | Other | Yes | 5º24'42.27''S; 48º21'53.96''W |
|  | MSc thesis | Other | Yes | 11º49'47.84''S; 50º04'22.90''W |
| [41] | Scientific literature | ISI | No | 3º 46'38.72"S; 46º8'48.17"W |
| [42] | Scientific literature | Google | No | 1º49'41.13''S; 53º23'51.98''W |
| [43] | MSc thesis | Google | No | 1°42.01'S; 48°54.01'W |
|  | MSc thesis | Google | No | 0°44'24.0"S; 48°30'17.2"W |
| [44] | Scientific literature | Google | Yes | 0º16'51.21''S; 48º22'30.03''W |
|  | Scientific literature | Google | Yes | 0º38'56.05''S; 47º30'01.63''W |
|  | Scientific literature | Google | Yes | 0º55'50.34''S; 48º18'11.75''W |
| [45] | Scientific literature | ISI | No | 2º45'15.76''S; 65º03'20.20''W |
| [46] | Scientific literature | ISI | No | 1º3'4.97''S; 48º5'2.93''W |
|  | Scientific literature | ISI | No | 1º3'7.34''S; 48º5'5.17''W |
|  | Scientific literature | ISI | No | 1º4'5.77''S; 48º5'9.83''W |
| [47] | Scientific literature | ISI | No | 4º02'57.47''S; 63º14'34.73''W |
|  | Scientific literature | ISI | No | 4º21'31.02''S; 63º24'28.50''W |
|  | Scientific literature | ISI | No | 4º11'19.69''S; 63º32'25.27''W |
|  | Scientific literature | ISI | No | 4º32'21.94''S; 64º54'14.89''W |
| [48] | Scientific literature | ISI | Yes | 0º57'26.88''S; 62º55'10.31''W |
|  | Scientific literature | ISI | Yes | 1º24'47.43''S; 61º57'43.62''W |
|  | Scientific literature | ISI | Yes | 0º33'23.83''S; 63º26'08.57''W |
|  | Scientific literature | ISI | Yes | 0º45'49.74''S; 63º02'22.86''W |
| [49] | PhD dissertation | Google | No | 1º25'54.86''S; 53º44'01.17''W |
|  | PhD dissertation | Google | No | 2º03'12.38''S; 54º14'15.40''W |
| [50] | Scientific literature | Google | No | 6°45'48.47"S; 67°40'46.85"W |
| [51] | MSc thesis | Other | No | 9°19'16.28"S; 68°12'32.22"W |
| [52] | Scientific literature | ISI | No | 7º41'15"S; 51º52'25"W |
| [53] | MSc thesis | Google | No | 1°16'18.13"S; 56°41'25.19"W |
| [54] | Scientific literature | ISI | No | 3°17'30.85"S; 55°11'22.60"W |
| [55] | PhD dissertation | Google | No | 2º53'59.16''S; 64º53'33.32''W |
| [56] | MSc thesis | Google | No | 1°14'8.80"N; 54°39'40.33"W |
| [57] | Chapter | Other | No | 1°55'20.52"S; 61°52'8.64"W |
| [58] | Scientific literature | ISI | No | 0°24'29.37"S; 60°31'13.63"W |
| [59] | Scientific literature | Google | No | 1º36'43.17''S; 49º13'24.64''W |
| [60] | MSc thesis | Other | No | 3º54'31.15''S; 52º50'01.61''W |
| [61] | MSc thesis | Other | No | 09º54'35.1''S; 49º59'35.4''W |
| [62] | Scientific literature | Google | No | 0°51'07.0''S; 46°36'02.5''W |
|  | Scientific literature | Google | No | 0°50'58.9''S; 46°36'28.7''W |
| [63] | Scientific literature | Google | No | 0º20'46.02''S; 63º45'04.95''W |
|  | Scientific literature | Google | No | 0º25'53.53''S; 65º00'16.14''W |
|  | Scientific literature | Google | No | 1º21'31.54''S; 61º58'49.46''W |
| [64] | Scientific literature | ISI | Yes | 2º07'42.32''S; 54º32'31.22''W |
|  | Scientific literature | ISI | Yes | 2º10'08.69''S; 54º45'05.75''W |
| [65] | PhD dissertation | Google | No | 2º4'59.99''S;  62º4'59.99''W |
| [66] | MSc thesis | Google | No | 13º01'23.51''S; 53º06'47.78''W |
|  | MSc thesis | Google | No | 12º25'41.13''S; 52º12'02.79''W |
|  | MSc thesis | Google | No | 11º04'18.63''S; 57º33'43.22''W |
|  | MSc thesis | Google | No | 12º12'37.79''S; 54º52'35.23''W |
|  | MSc thesis | Google | No | 10º16'26.31''S; 61º12'09.54''W |
|  | MSc thesis | Google | No | 11º25'50.34''S; 57º28'50.15''W |
| [67] | Chapter | Other | No | 3º43'21.57''S; 46º19'58.19''W |
| [68] | PhD dissertation | Google | No | 1º04'18.03''S; 46º45'15.23''W |
|  | PhD dissertation | Google | No | 2º16'10.94''S; 49º29'49.27''W |
|  | PhD dissertation | Google | No | 1º12'07.48''S; 47º10'37.81''W |
|  | PhD dissertation | Google | No | 1º17'20.68''S; 47º54'56.79''W |
|  | PhD dissertation | Google | No | 2º59'09.71''S; 47º21'11.23''W |
|  | PhD dissertation | Google | No | 1º49'41.13''S; 53º23'51.98''W |
|  | PhD dissertation | Google | No | 3º48'17.96''S; 49º46'23.07''W |
|  | PhD dissertation | Google | No | 3º43'15.44''S; 59º33'50.50''W |
|  | PhD dissertation | Google | No | 3º49'06.28''S; 60º21'40.42''W |
|  | PhD dissertation | Google | No | 3º08'01.74''S; 58º26'18.76''W |
|  | PhD dissertation | Google | No | 2º37'04.02''S; 60º56'46.48''W |
|  | PhD dissertation | Google | No | 1º26'47.36''S; 60º01'36.05''W |
|  | PhD dissertation | Google | No | 2º43'10.42''S; 59º40'50.92''W |
| [69] | Scientific literature | ISI | No | 4º28'44.40''S; 62º22'40.93''W |
|  | Scientific literature | ISI | No | 5º03'35.55''S; 62º29'03.81''W |
| [70] | MSc thesis | Other | No | 4º28'44.40''S; 62º22'40.93''W |
| [71] | Scientific literature | Google | No | 1º55'53.28''S; 61º52'07.78''W |
|  | Scientific literature | Google | No | 1º54'38.28''S; 61º26'35.16''W |
|  | Scientific literature | Google | No | 2º37'30.79''S; 60º55'08.21''W |
|  | Scientific literature | Google | No | 3º09'26.35''S; 60º01'49.03''W |
| [72] | Scientific literature | Google | No | 1º27'21''S; 48°30'16''W |
| [73] | Magazine | Google | No | 1º27'42.56''S; 48º27'47.99''W |
| [74] | Scientific literature | Google | No | 3º03'16.51''S; 64º50'48.34''W |
| [75] | Conference abstract | Other | No | 3º20'44.28''N; 64º42'54.77''W |
| [76] | Conference abstract | Other | No | 7º05'28.14''S; 60º59'32.89''W |
|  | Conference abstract | Other | No | 6º54'51,89''S;  62º35'50,80''W |
| [77] | MSc thesis | Other | No | 1º45'53.80''N; 51º05'53.21''W |
| [78] | Conference abstract | Other | No | 3º20'44.28''N; 64º42'54.77''W |
| [79] | Conference abstract | Other | No | 1º51'16.63''S; 49º18'43.41''W |
| [80] | Scientific literature | Google | No | 1º49'41.13''S; 53º23'51.98''W |
|  | Scientific literature | Google | No | 2º04'30.54''S; 54º36'18.60''W |
| [81] | MSc thesis | Other | No | 3º12'S; 64º35'W |
|  | MSc thesis | Other | No | 3º05'S; 64º57'W |
| [82] | Scientific literature | ISI | No | 2º10'44''S; 65º42'27.06''W |
| [83] | Conference abstract | Other | No | 4º24'20.30''S; 61º54'07.07''W |
| [84] | Conference abstract | Other | No | 4º24'20.30''S; 61º54'07.07''W |
| [85] | Conference abstract | Other | No | 3º20'44.28''N; 64º42'54.77''W |
| [86] | Conference abstract | Other | No | 2º10'44''S; 65º42'27.06''W |
| [87] | Conference abstract | Other | No | 2º10'44''S; 65º42'27.06''W |
| [88] | Conference abstract | Other | No | 2°31'49"S; 65°04'00"W |
| [89] | Conference abstract | Other | No | 2º10'44''S; 65º42'27.06''W |
| [90] | Conference abstract | Other | No | 4º24'20.30''S; 61º54'07.07''W |
| [91] | Conference abstract | Other | No | 2º58'25.02''S;  65º5'49.12''W |
| [92] | Conference abstract | Other | No | 2º10'44''S; 65º42'27.06''W |
| [93] | Conference abstract | Other | No | 3º56'24.93''S; 63º09'30.42''W |
| [94] | Conference abstract | Other | No | 3º20'44.28''N; 64º42'54.77''W |
| [95] | Conference abstract | Other | No | 3º20'38.25''S; 64º43'53.29''W |
| [96] | Scientific literature | ISI | No | 2°31'49"S; 65°04'00"W |
| [97] | Scientific literature | Google | No | 2º58'S; 55º50'W |
| [98] | PhD dissertation | Google | No | 2º4'59.99''S;  62º4'59.99''W |
| [99] | Conference abstract | Other | No | 2º10'44''S; 65º42'27.06''W |
| [100] | Conference abstract | Other | No | 3º34'37.81''S; 51º16'12.59''W |
| [101] | Conference abstract | Other | No | 0º52'19.18''N; 50º02'44.67''W |
| [102] | Conference abstract | Other | No | 4º6'34.95''S; 63º8'0.34''W |
| [103] | Conference abstract | Other | No | 4º6'34.95''S; 63º8'0.34''W |
| [104] | Conference abstract | Other | No | 2º10'44''S; 65º42'27.06''W |
| [105] | Conference abstract | Other | No | 3º20'44.28''N; 64º42'54.77''W |

**References**

1. Moura-Santos EA, Campos CEC, Almeida DF. Etnobiologia sobre a reprodução da tartaruga, *Podocnemis expansa* (Shweigger, 1812) em duas comunidades do Arquipélago do Bailique. Graduate Thesis, Universidade Federal do Amapá. 2011.

2. Arraes DRS, Cunha HFA, Tavares-Dias M. Nidificação, neonatos e a influência da pressão antrópica em tracajá *Podocnemis unifilis* TROSCHEL, 1848 (Podocnemididae) na bacia do Rio Araguari, Amazônia Oriental, Brasil. M.Sc. Thesis, Universidade Federal do Amapá. 2012. Available: http://www2.unifap.br/ppgbio/files/2010/05/DÉBORA-ARRAES-DISSERTAÇÃO-NIDIFICAÇÃO-NEONATOS-E-A-INFLUE.pdf

3. Von Mühlen EM, da Silveira R. Consumo de proteína animal em Aldeias de terra firme e de várzea da Terra Indígena Uaçá, Amapá, Brasil. M.Sc. Thesis, Universidade Federal do Pará. 2005. Available: http://repositorio.ufpa.br/jspui/handle/2011/4235

4. Norris D, Michalski F. Socio-economic and spatial determinants of anthropogenic predation on Yellow-spotted River Turtle, *Podocnemis unifilis* (Testudines: Pelomedusidae), nests in the Brazilian Amazon: Implications for sustainable conservation and management. Zoologia. 2013;30(5):482-90. doi: 10.1590/S1984-46702013000500003.

5. Barbosa DA, Silva CR, Lima DS. Conhecimento de moradores dos limites e entorno de sete Unidades de Conservação no estado de Amapá sobre a ocorrência e as possíveis ameaças ao Peixe-boi (*Trichechus* sp.). Graduate Thesis, Universidade Federal do Amapá. 2013.

6. da Silva DWS, Campos CEC. Percepções e conhecimento etnoherpetológico dos pais e filhos da comunidade da Ilha de Santana, município de Santana, Amapá. M.Sc. Thesis, Universidade Federal do Amapá. 2013.

7. de Melo CCS. Inventário biológico das áreas do Sucuriju e região dos Lagos, no Amapá. Mamíferos não Voadores da Região dos Lagos, Municípios de Tartarugalzinho, Pracuúba e Amapá, no Amapá. 21 ed. Macapá, Amapá. 2006. p. 196-217. Available: http://www.iepa.ap.gov.br/probio/relatorios/Relatorio_Cap13.pdf

8. Coutinho ME, Almeida de Andrade T, de Souza Lima F, Vieira Quaggio T, Vilhena Martins AA. Ecologia dos crocodilianos na Reserva Biológica do Lago Piratuba, Amapá. Goiânia, Goiás: Instituto Brasileiro do Meio Ambiente e dos Recursos Naturais Renováveis; Centro de Conservação e Manejo de Répteis e Anfíbios, 2007.

9. Conceição PCF, Kauano EE, Michalski F. Sustentabilidade em Unidades de Conservação: Avaliação do uso da fauna de vertebrados de médio e grande porte (com ênfase em quelônios) na Floresta Nacional do Amapá, Brasil. Graduate Thesis, Universidade Federal do Amapá. 2013. Available: http://www2.unifap.br/cambientais/files/2014/01/TCC_PC_10MAIO2013_FM14PM-2_Paula.pdf

10. da Silva CMN, Laufer J, Barboza RSL. Consumo de proteína de origem animal em comunidades da Reserva Biológica do Lago Piratuba, Amapá, Brasil. Graduate Thesis, Universidade Federal do Amapá. 2014. Available: http://www2.unifap.br/cambientais/files/2014/08/CONSUMO-DE-PROTEÍNA-DE-ORIGEM-ANIMAL-EM-COMUNIDADES-DA-RESERVA-BIOLÓGICA-DO-LAGO-PIRATUBA-–-AMAPÁ-BRASIL-.pdf

11. Luna FO, Lima RP, Araújo JP, Passavante JZO. Status de conservação do peixe-boi marinho (*Trichechus manatus manatus* Linnaeus, 1758) no Brasil. Revista Brasileira de Zoociências. 2008;10(2):145-53.

12. Cardoso E, Filho Rodrigues S, Costa J, Guedes M. Atividade de caça em uma comunidade na Reserva Extrativista do Rio Cajari, Amapá. XI Congresso de Ecologia do Brasil Biodiversidade e Sustentabilidade; 2013; Porto Seguro, Bahia.

13. Rebêlo GH, Pezzuti JCB, Lugli L, Moreira G. Pesca artesanal de Quelônios no Parque Nacional do Jaú (AM). Boletim do Museu Paraense Emilio Goeldi. 2005;1:111-27.

14. Waldez F, Gama e Adário L, Marioni B, Rossoni F, Erickson J. Monitoramento participativo da caça de quelônios (Podocnemididae) por comunitários ribeirinhos no baixo Rio Purus e proteção de sítios de desova na RSD Piagaçu-Purus, Brasil. Revista Colombiana de Ciencia Animal. 2013;5:4-23.

15. Iriarte V, Marmontel M. River Dolphin (*Inia geoffrensis*, *Sotalia fluviatilis*) Mortality Events Attributed to Artisanal Fisheries in the Western Brazilian Amazon. Aquatic Mammals. 2013;39(2):116-24. doi: 10.1578/AM.39.2.2013.116.

16. Iriarte V, Marmontel M. Insights on the use of dolphins (boto, *Inia geoffrensis* and tucuxi, *Sotalia fluviatilis*) for bait in the piracatinga (*Calophysus macropterus*) fishery in the western Brazilian Amazon. Journal of Cetacean Research and Management. 2013;13(2):163-73.

17. Mendonça CWS, da Silveira R. A caça comercial de jacarés no baixo rio purus e suas implicações no manejo sustentável na Reserva Piagaçu-Purus, Amazônia Central. M.Sc. Thesis, Universidade Federal do Amazonas. 2009. Available: http://www.ppgcasa.ufam.edu.br/pdf/dissertacoes/2009/Washington%20Carlos.pdf

18. da Silveira R, Thorbjarnarson JB. Conservation implications of commercial hunting of black and spectacled caiman in the Mamirauá Sustainable Development Reserve, Brazil. Biological Conservation. 1999;88(1):103-9. doi: 10.1016/S0006-3207(98)00084-6.

19. Alves LCPS, Zappes CA, Andriolo A. Conflicts between river dolphins (Cetacea: Odontoceti) and fisheries in the Central Amazon: a path toward tragedy? Zoologia (Curitiba). 2012;29(5):420-9. doi: 10.1590/S1984-46702012000500005.

20. Carvalho TS, Silva Jr. NJ. Manejo e uso sustentável do jacare tinga (*Caiman crocodilus*) por ribeirinhos: Um estudo avaliativo. M.Sc. Thesis, Pontifícia Universidade Católica de Goiás. 2011. Available: http://www.cpgss.pucgoias.edu.br/ArquivosUpload/2/file/MCAS/Thiago%20dos%20Santos%20Carvalho.pdf

21. Ataídes AG, Malvasio A, Parente TG. Percepções sobre o consumo de quelônios no entorno do Parque Nacional do Araguaia, Tocantins: conhecimentos para conservação. Gaia Scientia. 2010;4(1):7-20. doi: 1214-1409597402.

22. Luna FO, Araújo JP, Passavante JZO, Mendes PP, Pessanha M, Soavinski RJ, et al. Ocorrência do peixe-boi marinho (*Trichechus manatus manatus*) no litoral norte do Brasil. Boletim do Museu de Biologia Mello Leitão. 2008;23:37-49. Available: http://www.car-spaw-rac.org/IMG/pdf/Ocorrncia_de_peixe-boi_marinho_no_litoral_norte_do_Brasil.pdf

23. Valsecchi J, Valsecchi do Amaral P. Perfil da caça e dos caçadores na Reserva de Desenvolvimento Sustentável Amanã, Amazonas, Brasil. Uakari [Internet]. 2009; 5:[33-48 pp.]. Available: http://www.uakari.org.br/UAKARI/article/view/65/76.

24. Fachín-Terán A, Vogt RC, Thorbjarnarson JB. Padrões de caça e uso de quelônios na Reserva de Desenvolvimento Sustentável Mamirauá, Amazonas, Brasil. In: Cabrera E, Mercolli C, Resquin R, editors. Manejo de Fauna Silvestre en Amazonia y Latinoamérica. 1. Asunción, Paraguay 2000. p. 323-37.

25. Terra AK, Rebêlo GH. O uso da fauna pelos moradores da Comunidade São João e Colônia Central. In: Nelson E, Marques F, Vizoni V, Melo S, editors. Biotupé: Meio Físico, Diversidade Biológica e Sociocultural do Baixo Rio Negro, Amazônia Central. 1. INPA ed. Manaus, Amazonas: Instituto Nacional de Pesquisas da Amazônia; 2005. p. 141-53. Available: http://biotupe.org/livro/vol1/pdf/9_cap10.pdf

26. Lopes GP, Valsecchi J, Vieira TM, Amaral PV, Costa EWM. Hunting and hunters in lowland communities in the region of the middle Solimões, Amazonas, Brazil. Uakari [Internet]. 2012; 8:[7-18 pp.]. Available: http://www.uakari.org.br/UAKARI/article/view/120/167.

27. Franco DdL, Botero-Arias R, Marmontel M. A caça de jacarés para utilização como iscas para pesca da piracatinga na Reserva de Desenvolvimento Sustentável Mamirauá. XXIV Congresso brasileiro de zootecnia; 2014; Espírito Santo.

28. Arruda Campos MA, Santos GM, Py-Daniel V. Cruzando ecologias com os caçadores do Rio Cuieiras: saberes e estratégias de caça no baixo Rio Negro, Amazonas. M.Sc. Thesis, Universidade Federal do Amazonas. 2008. Available: www.iieb.org.br/index.php/download_file/939/268/

29. Alves LCPS, Andriolo A, Orams M, Azevedo ADF. Caracterização preliminar do comércio ilegal de animais silvestres na feira livre do Bairro da Liberdade, Manacapuru, Estado do Amazonas, Brasil. Sitientibus Série Ciências Biológicas. 10(2):236-43.

30. Belchior V, Andriolo A. Comunidades de seringueiros das Reservas Extrativistas do Rio Cautário, RO: Aspectos socioeconômicos, percepção ambiental e potenciais conflitos na interação com a fauna. M.Sc. Thesis, Universidade Federal Juiz de Fora. 2011. Available: http://www.ufjf.br/ecologia/files/2015/04/Dissertação_VeronicaBelchior.pdf

31. da Silva VMF, Martin AR, do Carmo NAS. Amazonian fisheries pose threat to elusive dolphin species. Magazine of the Species Survival Commission. 2011. Available: https://cmsdata.iucn.org/downloads/species_53_final.pdf

32. Pantoja-Lima J, Aride PHR, de Oliveira AT, Félix-Silva D, Pezzuti JC, Rebêlo GH. Chain of commercialization of *Podocnemis* spp. turtles (Testudines: Podocnemididae) in the Purus River, Amazon basin, Brazil: current status and perspectives. Journal of ethnobiology and ethnomedicine. 2014;10:10. doi: 10.1186/1746-4269-10-8. PubMed Central PMCID: PMC3933064.

33. Fuccio H, Carvalho EF, Vargas G. Perfil da caça e dos caçadores no Estado do Acre, Brasil. Revista Aportes Andinos. 2003;6:1-18. Available: http://www.insumisos.com/httpdocs/articulos/Perfil%20da%20ca%E7a%20e%20dos%20ca%E7adores%20no%20Estado%20do%20Acre.pdf

34. Alves RRN, Rosa IL. Use of Tucuxi Dolphin *Sotalia fluviatilis* for Medicinal and Magic/Religious Purposes in North of Brazil. Human Ecology 2008;36:443-7. doi: 10.1007/s10745-008-9174-5.

35. Alves RRN, Rosa IL. From cnidarians to mammals: the use of animals as remedies in fishing communities in NE Brazil. Journal of Ethnopharmacology. 2006;107(2):259-76. doi: 10.1016/j.jep.2006.03.007.

36. Mintzer VJ, Schmink M, Lorenzen K, Frazer TK, Martin AR, da Silva VMF. Attitudes and behaviors toward Amazon River dolphins (*Inia geoffrensis*) in a sustainable use protected area. Biodiversity and Conservation. 2014;24(2):247-69. doi: 10.1007/s10531-014-0805-4.

37. Aguilar CVC, Guimarães DE. Etnoconhecimento do peixe-boi amazônico (*Trichechus inunguis*): uso tradicional por ribeirinhos na Reserva Extrativista Tapajós Arapiuns e Floresta Nacional do Tapajós, Pará. M.Sc. Thesis, Universidade Federal do Pará. 2007. Available: http://repositorio.ufpa.br/jspui/handle/2011/4678

38. da Silva AL. Comida de gente: preferências e tabus alimentares entre os ribeirinhos do Médio Rio Negro (Amazonas, Brasil). Revista de Antropologia. 2007;50:125-79. doi: 10.1590/S0034-77012007000100004.

39. Parry L, Barlow J, Peres CA. Allocation of hunting effort by Amazonian smallholders: Implications for conserving wildlife in mixed-use landscapes. Biological Conservation. 2009;142(8):1777-86. doi: 10.1016/j.biocon.2009.03.018.

40. Salera Jr. G, Giraldin O, Malvasio A. Avaliação da biologia reprodutiva, predação natural e importância social em quelônios com ocorrência na bacia do Araguaia. M.Sc. Thesis, Fundação Universidade Federal do Tocantins. 2005. Available: http://static.recantodasletras.com.br/arquivos/1731432.pdf?1249959680

41. Prado HM, Forline LC, Kipnis R. Hunting practices among the Awá-Guajá: towards a long-term analysis of sustainability in an Amazonian indigenous community. Boletim do Museu Paraense Emilio Goeldi. 2012;7:479-91. doi: 10.1590/S1981-81222012000200010

42. Barboza RSL, Rebêlo GH, Pezzuti JCB, Barboza RSL. Plano de manejo comunitário de jacarés na várzea do baixo rio Amazonas,Santarém, PA, Brasil. Biotemas. 2013;26(2):215-26. doi: 10.5007/2175-7925.2013v26n2p215.

43. Rodrigues ALF, da Silva ML. O boto na verbalização de estudantes ribeirinhos: uma visão etnobiológica. M.Sc. Thesis, Universidade Federal do Pará. 2008. Available: http://repositorio.ufpa.br/jspui/handle/2011/5344

44. Brito T. O conhecimento ecológico local e a interação de botos com a pesca no litoral do estado do Pará, região Norte – Brasil. Biotemas. 2012;25(4):259-77. doi: 10.5007/2175-7925.2012v25n4p259.

45. Mintzer VJ, Martin AR, da Silva VMF, Barbour AB, Lorenzen K, Frazer TK. Effect of illegal harvest on apparent survival of Amazon River dolphins (*Inia geoffrensis*). Biological Conservation. 2013;158:280-6. doi: 10.1016/j.biocon.2012.10.006.

46. Baía-Júnior P, Guimarães DAA, Le Pendu Y. Non-legalized commerce in game meat in the Brazilian Amazon: a case study. Revista de Biología Tropical. 2010;58(3):1079-88. PubMed Central PMCID: PMC20737856.

47. Franzini AM, Castelblanco-Martínez DN, Rosas FCW, da Silva VMF. What do local people know about Amazonian manatees? Traditional ecological knowledge of Trichechus inunguis in the oil province of Urucu, AM, Brazil. Brazilian Journal of Nature Conservation. 2013;11(July 2013):75-80. doi: 10.4322/natcon.2013.012.

48. da Silva AL, Begossi A. Biodiversity, food consumption and ecological niche dimension: a study case of the riverine populations from the Rio Negro, Amazonia, Brazil. Environment, Development and Sustainability. 2009;11(3):489-507. doi: 10.1007/s10668-007-9126-z.

49. Barboza RSL, McGrath D, Pezzuti JCB. Etnoecologia, pesca e manejo comunitário de quelônios aquáticos na várzea do Baixo rio Amazonas. PhD Dissertation, Universidade Federal do Pará. 2012. Available: http://repositorio.ufpa.br/jspui/bitstream/2011/5985/1/Tese_EtnoecologiaPescaManejo.pdf

50. Pezzuti JCB, Chaves RP. Etnografia e manejo de recursos naturais pelos índios Deni, Amazonas, Brasil. Acta Amazonica. 2009;39(1):121-38. doi: 10.1590/S0044-59672009000100013.

51. Calouro AM, Marinho-Filho JS. Caça de subsistência: sustentabilidade e padrões de uso entre seringueiros ribeirinhos e não ribeirinhos do Estado do Acre. M.Sc. Thesis, Universidade de Brasilia. 1995. Available: http://www.pgeclunb.net.br/dissertacoes-defendidas/dissertacoes/1990-1999/414-armando-muniz-calouro-1995/file

52. Peres CA, Nascimento HS. Impact of game hunting by the Kayapó of south-eastern Amazonia: implications for wildlife conservation in tropical forest indigenous reserves. Biodiversity and Conservation. 2006;15(8):2627-53. doi: 10.1007/s10531-005-5406-9.

53. Melo LS, Giarrizzo T. Uso dos recursos alimentares por populações quilombolas da Amazônia brasileira. M.Sc. Thesis, Universidade Federal do Pará. 2012. Available: http://repositorio.ufpa.br/jspui/handle/2011/5987

54. Fonseca RA, Pezzuti CJB. Dietary breadth of the animal protein consumed by riverine communities in the Tapajós National Forest, Brazil. Revista de Biologia Tropical. 2013;61(1):263-72. PubMed Central PMCID: PMC23894979.

55. Valsecchi J, Figueira JEC. Caça de animais silvestres nas Reservas de Desenvolvimento Sustentável Mamirauá e Amanã. PhD Dissertation, Universidade Federal de Minas Gerais. 2012. Available: http://www.bibliotecadigital.ufmg.br/dspace/handle/1843/BUOS-978FZW

56. Van Velthem Linke IH, Mendes-Oliveira AC. Caracterização do uso da fauna cinegética em aldeias das etnias Wayana e aparai na Terra Indígena Parque do Tumucumaque, Pará. M.Sc. Thesis, Universidade Federal do Pará. 2009. Available: http://repositorio.ufpa.br/jspui/handle/2011/4439

57. Pezzuti JCB, Rebêlo GH, da Silva DF, Pantoja Lima J, Correa Ribeiro M. A Caça e a Pesca no Parque Nacional do Jaú. In: INPA, editor. Janelas para a biodiversidade no Parque Nacional do Jaú: Uma estratégia para o estudo da biodiversidade na Amazônia. 1. Fundação Vitória Amazônica ed. Manaus, Amazonas 2004. p. 213-30.

58. Souza-Mazurek RR, Pedrinho T, Feliciano X, Hilário W, Gerôncio S, Marcelo E. Subsistence hunting among the Waimiri Atroari Indians in central Amazonia, Brazil. Biodiversity and Conservation. 2000;9(5):579-96. doi: 10.1023/A:1008999201747.

59. Ribeiro ASS, Palha MDD, Tourinho MM, Whiteman C, Silva ADS. Utilização dos recursos naturais por comunidades humanas do Parque Ecoturístico do Guamá, Belém, Pará. Acta Amazonica. 2007;37(2):235-40. doi: 10.1590/S0044-59672007000200009.

60. Castro RRA, Oliveira MCC. Comunidades Tradicionais e Unidades de Conservação no Pará: A influência da criação da Reserva Extrativista Rio Xingu, Terra do Meio, nos modos de vida das famílias locais. M.Sc. Thesis, Universidade Federal do Pará. 2013. Available: http://mafds.websimples.info/files/arquivo/156/roberta-rowsy.pdf

61. Ataídes AG, Malvásio A. Parâmetros populacionais, aspectos reprodutivos e importância socioeconômica de *Podocnemis unifilis* (TROSCHEL,1848) (Testudines, Podocnemididae), no entorno do Parque Nacional do Araguaia, Tocantins. M.Sc. Thesis, Universidade Federal do Tocantins. 2009. Available: http://livros01.livrosgratis.com.br/cp126095.pdf

62. Barboza RSL, Barboza MSL, Pezzuti JCB. Aspectos culturais da zooterapia e dieta alimentar de pescadores artesanais do litoral paraense. Fragmentos de Cultura. 2014;24(2):253-66.

63. da Silva AL. Animais medicinais: conhecimento e uso entre as populações ribeirinhas do rio Negro, Amazonas, Brasil. Boletim do Museu Paraense Emílio Goeldi. 2008;3(3):343-57. doi: 10.1590/S1981-81222008000300005.

64. Miorando PS, Rebêlo GH, Teófilo Pignati M, Pezzuti JCB. Effects of community-based management on Amazon river turtles: A case study of Podocnemis sextuberculata in the lower Amazon floodplain, Pará, Brazil. Chelonian Conservation and Biology. 2013;12(1):143-50. doi: 10.2744/CCB-1011.1.

65. Pezzuti JCB, Begossi A. Ecologia e etnoecologia de quelônios no Parque Nacional do Jaú, Amazonas, Brasil. PhD Dissertation, Universidade Estadual de Campinas. 2003. Available: http://www.bibliotecadigital.unicamp.br/document/?code=vtls000311253

66. Crepaldi GB, Januário ERS. Alimentação indígena em Mato Grosso: educação ambiental e sustentabilidade entre etnias de estudantes da faculdade indígena intercultural. M.Sc. Thesis, Universidade do Estado de Mato Grosso. 2012. Available: http://www.unemat.br/prppg/ppgca/docs2012/gabrielle_crepaldi.pdf

67. Martins MB, de Oliveira TG. Utilização de caça pelos índios Awá/Guajá e Ka’apor da Amazônia Maranhense. In: Martins MB, Oliveira TG, editors. Amazônia Maranhense: diversidade e conservação. 1. Belém, Pará: Museu Paraense Emílio Goeldi; 2011. p. 271-9.

68. Mendes FLS, Simonian LTL. Ilegalidade no comércio de animais silvestres nos estados de Pará e Amazonas. PhD Dissertation, Universidade Federal do Pará. 2010. Available: http://repositorio.ufpa.br/jspui/handle/2011/2715

69. de Souza DA, da Silva VMF, Silva JCF, Muhlen E, Antunes A, Rossoni F. Conservation prospects for the Amazonian manatee in the lower Purus River, Central Amazon, Brazil. SireNews: Newsletter of the IUCN Sirenia Specialist Group. 2014;62:6-8.

70. Vieira MARM, Shepard GH. Influências dos sistemas de manejo formal e informal na atividade de caça de subsistência na RDS Piagaçu-Purus, AM. M.Sc. Thesis, Instituto Nacional de Pesquisas da Amazônia. 2013. Available: http://www.piagacu.org.br/sites/default/files/dissertacao_marinavieira.pdf

71. Rebêlo G, Pezzuti JCB. Percepções sobre o consumo de quelônios na Amazônia. Sustentabilidade e alternativas ao manejo atual. Ambiente e Sociedade. 2000;6:85-104. doi: 10.1590/S1414-753X2000000100005.

72. Figueiredo N. Os “bichos” que curam: os animais e a medicina de “folk” em Belém do Pará. Boletim do Museu Paraense Emilio Goeldi. 1994;10(1):75-91.

73. Bitencourt BLG, Costa Lima PG, Barros FB. Comércio e uso de plantas e animais de importância mágico religiosa e medicinal no mercado público do guama, Belém do Pará. Revista Faculdade Santos Agostino. 2014:96-158.

74. Botero-Arias R, Marmontel M, Queiroz HL. Projeto de manejo experimental de jacarés no estado do Amazonas: Abate de jacarés no setor Jarauá, Reserva de Desenvolvimento Sustentável Mamirauá, dezembro de 2008. Uakari [Internet]. 2009; 5:[49-58 pp.]. Available: http://www.uakari.org.br/UAKARI/article/view/66/77.

75. Lopes VL, El Bizri HR, Morcatty TQ, Valsecchi J. O comércio e a procedência da carne de caça no mercado municipal de Tefé, Amazonas, Brasil. Livro de Resumos 11º Simpósio sobre Conservação e Manejo Participativo na Amazônia; 2014; Tefé, Amazonas.

76. Pantoja TMdA, Queiroz HL, Kendall S. Análise de dados de conhecimento local, sítios de ocorrência e conservação do peixe-boi da amazônia (Trichechus inunguis NATTERER, 1883) no baixo Javari, AM, Brasil. Livro de Resumo 11º Simpósio sobre Conservação e Manejo Participativo na Amazônia; 2014; Tefé, Amazonas.

77. Ribeiro ABN, Santos C. Captura e implicações da pressão antrópica para o tracajá (*Podocnemis unifilis* TROSCHEL, 1848) na região dos lagos do município de Pracuúba, Amazônia, Brasil. M.Sc. Thesis, Universidade Federal do Amapá. 2012. Available: http://www2.unifap.br/ppgbio/files/2010/05/ANA-BEATRIZ-NUNES-RIBEIRO1.pdf

78. Brum S. Estimates for *Inia geoffrensis* mortality used as bait in piracatinga fishery in Central Amazon‚ Brazil. Libro de resúmenes La biodiversidad sensible: Un patrimonio natural irreemplazable; 2014; Cartagena de Indias, Colombia.

79. Ristau N, Farias J, Marmontel M, Trujillo F. Interações do boto Inia araguaiaensis com a pesca na bacia Tocantins-Araguaia‚ Brasil. Libro de resúmenes La biodiversidad sensible: Un patrimonio natural irreemplazable; 2014; Cartagena de Indias, Colombia.

80. Barboza RSL, Barboza MSL, Pezzuti JCB. Estava pescando de malhadeira, vi na praia uns cascos brilhando, era luar, abeirei a terra e fui pegar: Práticas de pesca de quelônios na varzea amazonica (Santarém, PA). Amazônica-Revista de Antropologia. 2013;5(3):622-53.

81. Brum SM, da Silva VMF. Interação dos golfinhos da Amazônia com a pesca no médio Solimões. M.Sc. Thesis, Instituto Nacional de Pesquisas Da Amazônia. 2011. Available: http://bdtd.inpa.gov.br/handle/tede/1439

82. Loch C, Marmontel M, Simões-Lopes PC. Conflicts with fisheries and intentional killing of freshwater dolphins (Cetacea: Odontoceti) in the Western Brazilian Amazon. Biodiversity and Conservation. 2009;18:3979-88. doi: 10.1007/s10531-009-9693-4.

83. Waldez F, Gama LA, Marioni B, Rossoni F. Monitoramento participativo da caça de quelônios (Podocnemididae) por comunitarios ribeirinhos no baixo Rio Purus e proteção de sitios de desova na RSD Piagaçu-Purus. Livro de Resumos 9º Seminário Anual de Pesquisa; 2012; Tefé, Amazonas.

84. Cal G, Marioni B. Monitoramento das populacoes de jacarés (*Melanosuchus niger* e *Caiman crocodilus crocodilus*) e da caça ilegal praticada na RSD Piagaçu-Purus. Livro de Resumos 9º Seminário Anual de Pesquisa; 2012; Tefé, Amazonas.

85. Santos Júnior LC, Souza LL. Diagnóstico de uso de fauna cinegética no municipio de Tefé, Amazonas. Livro de Resumos 9º Seminário Anual de Pesquisa; 2012; Tefé, Amazonas.

86. Iriarte V, Marmontel M. Mortalidade de golfinhos (Inia georensis, Sotalia uviatilis) associada a atividades de pesca no baixo Rio Japurá. Livro de Resumos 9º Seminário Anual de Pesquisa; 2012; Tefé, Amazonas.

87. Valsecchi J. Padrões de caça nas Reservas de Desenvolvimento Sustentável Mamirauá e Amanã. Livro de Resumo 10º Simpósio sobre Conservação e Manejo Participativo na Amazônia; 2013; Tefé, Amazonas.

88. Dutra JCO. A relação entre o poder do encantado e a interação dos moradores da Ti Cuiu-Cuiu com os botos vermelhos (*Inia geoffrensis*). Livro de Resumo 10º Simpósio sobre Conservação e Manejo Participativo na Amazônia; 2013; Tefé, Amazonas.

89. Torralvo K, Botero-Arias R. Predação de ninhos de jacarés na Reserva de Desenvolvimento Sustentável Mamirauá, AM. Livro de Resumos 10º Simpósio sobre Conservação e Manejo Participativo na Amazônia; 2013; Tefé, Amazonas.

90. Brum SM, da Silva VMF. Manejo participativo de pesca: Importante ferramenta para a conservação dos golfinhos da Amazônia. Livro de Resumo 10º Simpósio sobre Conservação e Manejo Participativo na Amazônia; 2013; Tefé, Amazonas.

91. Iriarte V, Marmontel M. Ameaças aos golfinhos amazônicos (*Inia geoffrensis*, *Sotalia fluviatilis*) no Baixo Rio Japurá. Livro de Resumos 10º Simpósio sobre Conservação e Manejo Participativo na Amazônia; 2013; Tefé, Amazonas.

92. Pires CM, Botero-Arias R. Conflito entre comunidades ribeirinhas e jacarés em dois setores da Reserva de Desenvolvimento Sustentável Mamirauá no período da enchente de 2012. Livro de Resumos 10º Simpósio sobre Conservação e Manejo Participativo na Amazônia; 2013; Tefé, Amazonas.

93. Ribeiro JES, Cota MRC, Lopes GP, Valsecchi J. Levantamento do mercado e preço da fauna cinegética na cidade de Coari, Amazonas, Brasil. Livro de Resumos 10º Simpósio sobre Conservação e Manejo Participativo na Amazônia; 2013; Tefé, Amazonas.

94. da Silva JA, Botero-Arias R, Santos Camillo C. Aspectos do comércio de quelônios no município de Tefé, AM. Livro de Resumos 10º Simpósio sobre Conservação e Manejo Participativo na Amazônia; 2013; Tefé, Amazonas.

95. Santos Júnior LC, Marmontel M. Mortalidade de botos amazônicos (Cetacea, Odontoceti) no município de Tefé, Amazonas. Livro de Resumos 10º Simpósio sobre Conservação e Manejo Participativo na Amazônia; 2013; Tefé, Amazonas.

96. Dutra JCO, Santos RBC. Enchantment experiences and the relation between the Miraña of Cuiú-Cuiú and the Pink River Dolphin (*Inia geoffrensis*). Uakari [Internet]. 2014; 10:[1-18 pp.]. Available: http://www.uakari.org.br/UAKARI/article/view/152/203.

97. Murrieta RSS. Dialética do sabor: alimentação, ecologia e vida cotidiana em comunidades ribeirinhas da Ilha de Ituqui, Baixo Amazonas, Pará. Revista de Antropología. 2001;44:39-88. doi: 10.1590/S0034-77012001000200002

98. Rebêlo GH, Abe AS. Quelônios, jacares e ribeirinhos no Parque Nacional do Jau (AM). PhD Dissertation, Universidade Estadual de Campinas. 2002. Available: http://www.bibliotecadigital.unicamp.br/document/?code=vtls000241014

99. Franco DL, Botero-Arias R, Marmontel M. Aspectos produtivos da pesca da Piracatinga (Calophysus macropterus) na Reserva de Desenvovimento Sustentável Mamirauá, medio Solimões, Amazonas. Livro de Resumo 11º Simpósio sobre Conservação e Manejo Participativo na Amazônia; 2014; Tefé, Amazonas.

100. Barbosa DA, Lima DS, da Silva CR, Marmontel M. Conhecimento de moradores dos limites e entorno de sete unidades de conservação no estado do Amapá sobre a ocorrência e as ameaças aos peixes-bois (*Trichechus* spp.). Livro de Resumo 11º Simpósio sobre Conservação e Manejo Participativo na Amazônia; 2014; Tefé, Amazonas.

101. Barbosa DA, Lima D, Marmontel M. Ameaças aos cetáceos amazônicos na região costeira do estado do Amapá. Livro de Resumo 11º Simpósio sobre Conservação e Manejo Participativo na Amazônia; 2014; Tefé, Amazonas.

102. Marreira JG, Marmontel M, Botero-Arias R, Falcão CM. Resultados preliminares da pesca da Piracatinga (Calophysus macropterus) na região de Coari, Médio Solimões. Livro de Resumo 11º Simpósio sobre Conservação e Manejo Participativo na Amazônia; 2014; Tefé, Amazonas.

103. Ribeiro JES, Lopes GP, Cota MR, Valsecchi J. Abastecimento do mercado de caça na cidade de Coari – Amazonas, Brasil. Livro de Resumo 11º Simpósio sobre Conservação e Manejo Participativo na Amazônia; 2014; Tefé, Amazonas.

104. Torralvo K, Botero-Arias R. Predação em ninhos de jacarés nas Reservas de Desenvolvimento Sustentável Mamirauá e Amanã. Livro de Resumos 11º Simpósio sobre Conservação e Manejo Participativo na Amazônia; 2014; Tefé, Amazonas.

105. Pimenta NC, Botero-Arias R, Marmontel M. Caracterização da atividade de pesca de Piracatinga (*Calophysus macropterus*) na Reserva de Desenvolvimento Sustentável Mamirauá, Amazonas, Brasil. Livro de Resumo 11º Simpósio sobre Conservação e Manejo Participativo na Amazônia; 2014; Tefé, Amazonas.
