# Supplementary material for: Implications of Scientific Collaboration Networks on Studies of Aquatic Vertebrates in the Brazilian Amazon
Source: PLoS One. 2016 Jun 28;11(6):e0158413. doi: 10.1371/journal.pone.0158413 (PMC4924867; doi:10.1371/journal.pone.0158413)
Supplement: S2 Table — (DOC) [file pone.0158413.s002.doc]

**S2 Table. List of research institutes/universities that conducted studies on Amazonian aquatic vertebrates.**

List of full name, abbreviation and website of research institutes/universities from the affiliation of first and last authors from 105 studies on subsistence and/or commercial hunting of Amazonian aquatic vertebrates from 1990 to 2014.

| Institution Full Name | Institution Abbreviation | Website |
| --- | --- | --- |
| Empresa Brasileira de Pesquisa Agropecuária | EMBRAPA | <https://www.embrapa.br/> |
| Fundação Nacional do Índio | FUNAI | <http://www.funai.gov.br/> |
| Instituto Brasileiro do Meio Ambiente e dos Recursos Naturais Renováveis | IBAMA | <http://www.ibama.gov.br/> |
| Instituto Chico Mendes de Conservação da Biodiversidade | ICMBio | [www.icmbio.gov.br/](http://www.icmbio.gov.br/) |
| Instituto de Desenvolvimento Sustentável Mamirauá | IDSM | <http://www.mamiraua.org.br/> |
| Instituto de Pesquisa Cientifica e Tecnológica do Estado do Amapá | IEPA | <http://www.iepa.ap.gov.br/> |
| Instituto Federal de Educação, Ciência e Tecnologia do Amazonas | IFAM | <http://www2.ifam.edu.br/campus/cmc> |
| Instituto Federal do Pará | IFPA | <http://www.ifpa.edu.br/> |
| Instituto Nacional de Pesquisas da Amazônia | INPA | <http://portal.inpa.gov.br/> |
| Instituto de Pesquisa Ambiental da Amazônia | IPAM | <http://www.ipam.org.br/> |
| Instituto Piagaçu | IPI | <http://www.piagacu.org.br/> |
| Fundación Omacha | OMACHA | <http://www.omacha.org/> |
| Sociedade Brasileira de Espeleologia | SBE | <http://www.cavernas.org.br/> |
| Pontifícia Universidade Católica de Goiás | PUC Goiás | <http://sites.pucgoias.edu.br/home/> |
| Universidade do Estado do Amazonas | UEA | <http://www2.uea.edu.br/> |
| Universidade Estadual do Maranhão | UEMA | <http://www.uema.br/> |
| Universidade Estadual do Pará | UEPA | <http://www.uepa.pa.gov.br/> |
| Universidade Estadual de Paraíba | UEPB | <http://www.uepb.edu.br/> |
| Universidade do Estado do Rio de Janeiro | UERJ | <http://www.uerj.br/> |
| Universidade Federal do Acre | UFAC | <http://www.ufac.br/> |
| Universidade Federal do Amazonas | UFAM | <http://www.ufam.edu.br/> |
| Universidade Federal de Juiz de Fora | UFJF | <http://www.ufjf.br/portal/> |
| Universidade Federal de Lavras | UFLA | <http://www.ufla.br/> |
| Universidade Federal de Minas Gerais | UFMG | <https://www.ufmg.br/> |
| Universidade Federal do Oeste do Pará | UFOPA | <http://www.ufopa.edu.br/> |
| Universidade Federal do Pará | UFPA | <http://www.portal.ufpa.br/> |
| Universidade Federal de Pernambuco | UFPE | <https://www.ufpe.br/ufpenova/> |
| Universidade Federal Rural da Amazônia | UFRA | <https://portal.ufra.edu.br/> |
| Universidade Federal de Santa Catarina | UFSC | <http://ufsc.br/> |
| Universidade Federal do Tocantins | UFT | <http://ww1.uft.edu.br/> |
| Universidade de Brasília | UNB | <http://www.unb.br/> |
| Universidade do Estado de Mato Grosso | UNEMAT | <http://www.novoportal.unemat.br/> |
| Universidade Estadual de São Paulo | UNESP | <http://www.unesp.br/> |
| Universidade Estadual de Campinas | UNICAMP | <http://www.unicamp.br/unicamp/> |
| Universidade Católica de Pernambuco | UNICAP | <http://www.unicap.br/home/> |
| Universidade Federal do Amapá | UNIFAP | <http://www.unifap.br/public/> |
| Universidade Paulista | UNIP | <http://www.unip.br/> |
| Universidade de São Paulo | USP | <http://www5.usp.br/> |
| University of Florida | UFL | <http://www.ufl.edu/> |
| Universidad Nacional Mayor de San Marcos | UNMSM | <http://www.unmsm.edu.pe/> |
| Universidade de Lisboa | ULISBOA | <http://www.ulisboa.pt/> |
| University of East Anglia | UEA-UK | <https://www.uea.ac.uk/> |
| Wildlife Conservation Society | WCS | <http://www.wcs.org/> |
